# Stretchable origami robotic arm with omnidirectional bending and twisting

**DOI:** 10.1073/pnas.2110023118

**Published:** 2021-08-30

**Authors:** Shuai Wu, Qiji Ze, Jize Dai, Nupur Udipi, Glaucio H. Paulino, Ruike Zhao

**Affiliations:** ^a^Department of Mechanical and Aerospace Engineering, The Ohio State University, Columbus, OH 43210;; ^b^School of Civil and Environmental Engineering, Georgia Institute of Technology, Atlanta, GA 30332

**Keywords:** origami robotic arm, magnetic actuation, omnidirectional bending, multimodal deformation

## Abstract

Inspired by the embodied intelligence observed in octopus arms, we introduce magnetically controlled origami robotic arms based on Kresling patterns for multimodal deformations, including stretching, folding, omnidirectional bending, and twisting. The highly integrated motion of the robotic arms is attributed to inherent features of the reconfigurable Kresling unit, whose controllable bistable deploying/folding and omnidirectional bending are achieved through precise magnetic actuation. We investigate single- and multiple-unit robotic systems, the latter exhibiting higher biomimetic resemblance to octopus’ arms. We start from the single Kresling unit to delineate the working mechanism of the magnetic actuation for deploying/folding and bending. The two-unit Kresling assembly demonstrates the basic integrated motion that combines omnidirectional bending with deploying. The four-unit Kresling assembly constitutes a robotic arm with a larger omnidirectional bending angle and stretchability. With the foundation of the basic integrated motion, scalability of Kresling assemblies is demonstrated through distributed magnetic actuation of double-digit number of units, which enables robotic arms with sophisticated motions, such as continuous stretching and contracting, reconfigurable bending, and multiaxis twisting. Such complex motions allow for functions mimicking octopus arms that grasp and manipulate objects. The Kresling robotic arm with noncontact actuation provides a distinctive mechanism for applications that require synergistic robotic motions for navigation, sensing, and interaction with objects in environments with limited or constrained access. Based on small-scale Kresling robotic arms, miniaturized medical devices, such as tubes and catheters, can be developed in conjunction with endoscopy, intubation, and catheterization procedures using functionalities of object manipulation and motion under remote control.

## Significance

The octopus quickly reconfigures its arms to perform highly integrated tasks, such as swimming, walking, and preying. Inspired by such a soft-bodied cephalopod biosystem, we engineer compliant origami robotic arms to achieve multimodal deformations that integrate stretching, folding, omnidirectional bending, and twisting for functions such as grasping and lifting objects by means of precise magnetic actuation. The remote magnetic field control allows distributed actuation of the multiple degree-of-freedom robotic system for complex motions to achieve the aforementioned shape-changing capabilities and functionalities. Origami robotic arms with untethered control are applicable to biomedical devices and morphing mechanisms in environments with limited access.

Compared to traditional robotic arms, where rigid links are connected by joints to provide rotational and translational degrees of freedom (DOFs), the soft counterparts in cephalopods—for example, octopus arms—exhibit intriguing features such as large and continuous deformations, adjustable compliance, and agile motions for moving and preying (1). Inspired by such biosystems, compliant mechanisms like foldable origami have been explored, as they allow reshaping of planar materials or structures into intricate three-dimensional (3D) architectures in various scales for robotic motions (2, 3) that can be applied to engineering fields including morphing structures (4–7), biomedical devices (8, 9), aerospace (10, 11), and electronics (12–14). Different origami mechanisms for robotic arms have been studied to achieve motions such as contraction (15, 16), deployment (17–19), bending (20, 21), and twisting (22, 23). These motions have been demonstrated for various functions—for instance, object grasping and biopsy (24–28). However, most existing origami robotic arms’ motions are hindered by limited DOFs, such as contraction/deployment-only (29), single-directional bending (30), and bidirectional bending (31). Although some systems have been developed to have limited integrated motions with multiple DOFs, they generally rely on multiple bulky actuators and/or wired driving forces—for example, motors (22, 23, 32) and pneumatic pumps (33)—which significantly limit the operation flexibility and versatility of the robotic arm in harsh environments with limited human and machine access. Motivated by these existing problems, a remotely actuated origami mechanism that can provide agile multi-DOF deformation for integrated large contraction/deployment, omnidirectional bending, and twisting is highly desired.

**Fig. 1.**
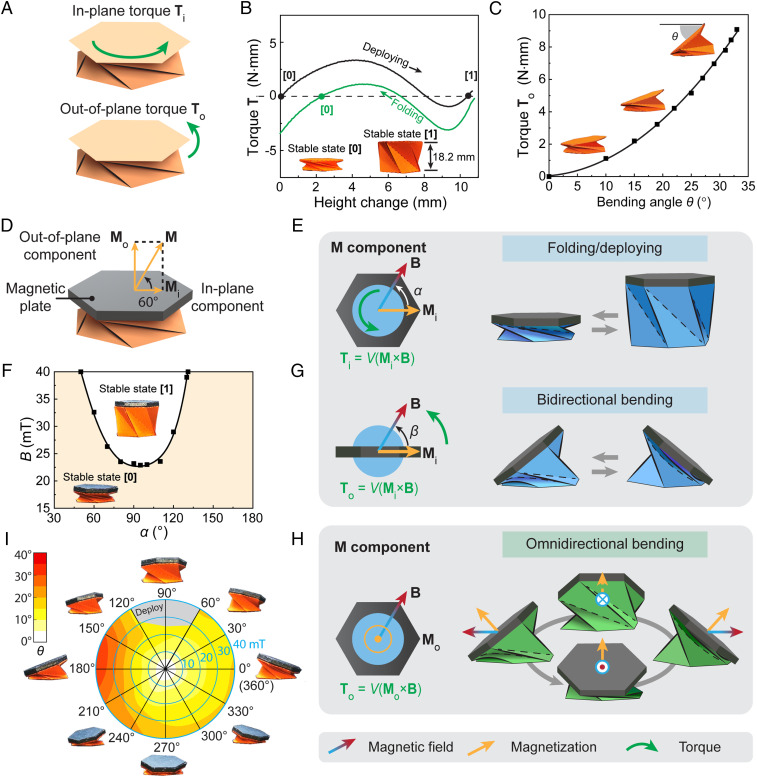
Actuation mechanisms of magnetic Kresling unit for folding/deploying, bidirectional bending, and omnidirectional bending. (*A*) Folding/deploying and bending deformation modes of Kresling origami induced by in-plane and out-of-plane torques, respectively. (*B*) Mechanical characterization of the folding and deploying processes. Images show the folded unit (stable state [0]) and deployed unit (stable state [1]). (*C*) Mechanical characterization of the bending behavior. Dots are from experimental measurements, fitted by a polynomial function. *C*, *Insets* are images of a unit with different bending angles. (*D*) Magnetic Kresling with designed inclined magnetization (60°) for both folding/deploying and omnidirectional bending. (*E*) Actuation mechanism of folding/deploying induced by the in-plane magnetization $M_{i}$ and in-plane magnetic field B. (*F*) Phase diagram showing the magnetic field conditions for switching the Kresling unit with inclined magnetization from the folded state (stable state [0]) to the deployed state (stable state [1]). Dots are from experimental measurements, fitted by a polynomial function. (*G*) Actuation mechanism of bidirectional bending induced by the in-plane magnetization $M_{i}$ and out-of-plane magnetic field B. (*H*) Actuation mechanism of omnidirectional bending induced by the out-of-plane magnetization $M_{o}$ and in-plane magnetic field B. (*I*) Polar plot and experimental images showing the bending angles in all directions. The applied magnetic field is perpendicular to the axial direction of the undeformed unit with inclined magnetization. The gray area denotes the conditions when the folded unit deploys.

Kresling origami, created from buckling of thin shell cylinders (34, 35), is an ideal building block for the origami robotic arm due to its inherent capability of multimodal deformation that provides deploying/folding and bending. As shown in Fig. 1*A*, the folding/deploying is induced by an in-plane torque $T_{i}$, and the bending is induced by an out-of-plane torque $T_{o}$. In-plane and out-of-plane are defined with respect to the plane of the undeformed hexagonal plane. The bistable Kresling origami with the stable folded state [0] and the stable deployed state [1] is achieved by geometrical design (36) (*SI Appendix*, Fig. S1). Under application of a positive in-plane torque (counterclockwise direction), the folded unit (stable state [0]) gradually deploys with the increased torque and snaps to the stable state [1] after it overcomes the energy barrier (Fig. 1*B*). Similarly, the deployed unit can fold back to the stable state [0] under a negative torque (*SI Appendix*, Fig. S2). When an out-of-plane torque is applied to the top hexagon of the Kresling unit, it bends with an angle of θ, defined as the angle between the horizontal direction and the top hexagon (Fig. 1*C* and *SI Appendix*, Fig. S3). The bending angle increases with the applied out-of-plane torque and has a maximum value due to the geometric constraints of the pattern. As discussed above, the direction and plane of the applied torque together determine the Kresling origami’s deformation mode: folding/deploying or bending. To realize fast-switchable deformation modes, we introduce magnetic actuation (37–40) to effectively and remotely control the instantaneous shift of the torque direction and torque plane for highly integrated complex motions, which haven’t been achieved by conventional actuation strategies (31, 32). With delicate designs of the magnetic Kresling structures and precise controls of the applied magnetic field, origami robotic arms with integrated multimodal deformations for large contraction/deployment, omnidirectional bending, and twisting are demonstrated in the following sections. Meanwhile, magnetic actuation enables small-scale robotic arms with the capability of flexible omnidirectional bending and integrated motions, allowing for the development of miniaturized medical devices in confined biomedical environments, such as stomach, intestine, trachea, and bronchi.

## Results and Discussion

### Multimodal Deformation of Kresling Unit under Magnetic Actuation.

Here, we use magnetic actuation to provide the torques for both folding/deploying and omnidirectional bending of the Kresling unit by simply attaching a magnetic plate to its top plane and actuating it under well-controlled 3D magnetic fields (36) (Movie S1 and *SI Appendix*, Fig. S4). A magnetic torque is generated when the magnetization of the magnetic plate tries to align itself with the applied magnetic field. Once the magnetization is set, the direction and intensity of the resultant torque can be controlled by tuning the direction and intensity of the applied magnetic field. Fig. 1*D* shows an example of the magnetic Kresling unit with an inclined magnetization that is 60° with respect to the Kresling unit’s top plane. In this case, the Kresling unit can provide both folding/deploying and omnidirectional bending under different programmed magnetic fields B, as the magnetization M has both in-plane magnetization component $M_{i}$ and out-of-plane magnetization component $M_{o}$. Fig. 1*E* illustrates the mechanism of folding/deploying behavior of the Kresling unit, which is attributed to the in-plane magnetization $M_{i}$ under an in-plane magnetic field B, with the initial angle between $M_{i}$ and B to be α. It results in an in-plane torque $T_{i}=\text{V}\left(M_{i}\times B\right)$ to fold or deploy the unit (Movie S1), where V is the volume of the magnetic plate. The phase diagram in Fig. 1*F* dictates the experimental conditions for the Kresling unit to deploy from the stable state [0] (orange region) to the stable state [1] (white region), where B is the intensity of B. Additionally, the magnetic field conditions for the Kresling unit to fold from the stable state [1] to the stable state [0] are shown in the *SI Appendix*, Fig. S5. The in-plane magnetization $M_{i}$ can also provide bidirectional bending under an out-of-plane magnetic field B, as shown in Fig. 1*G*. When B is applied with an initial angle of β to $M_{i}$, the out-of-plane torque $T_{o}=\text{V}\left(M_{i}\times B\right)$ leads to bidirectional bending of the Kresling unit (Fig. 1*G* and Movie S1). The omnidirectional bending deformation relies on an out-of-plane magnetization component $M_{o}$, as shown in Fig. 1*H*. Since out-of-plane magnetic torques $T_{o}=\text{V}\left(M_{o}\times B\right)$ can be generated by B in any directions that are not aligned with $M_{o}$, the bending direction of the Kresling unit is determined by the field direction, which is omnidirectional (Movie S1). It should be noted that if the magnetic plate only possesses out-of-plane magnetization, the bending angle is homogeneous under the applied in-plane sweeping magnetic field. With the 60° inclined magnetization (coupled in-plane and out-of-plane magnetizations), the omnidirectional bending angle of the Kresling unit is shown in Fig. 1*I*. Note that the in-plane magnetization component $M_{i}$ could affect the bending angle when the in-plane B sweeps with a constant intensity (10 to 40 mT with a step of 10 mT). With an increasing magnetic field intensity, θ increases in all directions, but the bending toward 180° is higher due to the influence of $M_{i}$. When the applied magnetic field further increases and the in-plane magnetic torque reaches a critical value, the unit may deploy under certain magnetic field conditions denoted by the gray region in Fig. 1*I*. The inhomogeneous bending angle can be compensated by applying a varying magnetic field. Additionally, the bending angle can be enlarged by adjusting the angle between $M_{o}$ and B (*SI Appendix*, Fig. S6).

### Integrated Motion of Omnidirectional Bending and Deploying.

**Fig. 2.**
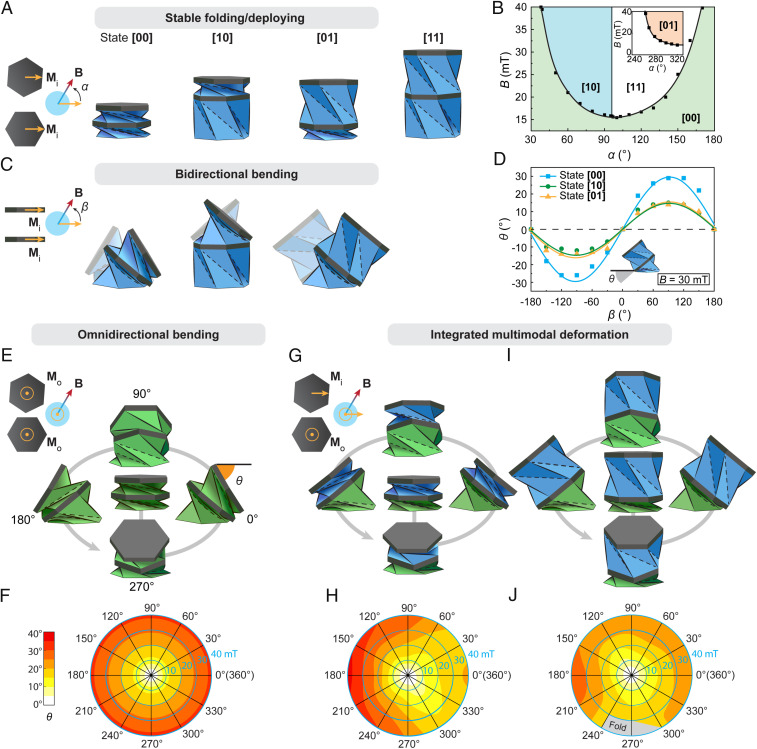
Integrated motion of two-unit magnetic Kresling assemblies. (*A*) State shifting of a two-unit Kresling assembly with in-plane magnetizations $M_{i}$ and in-plane magnetic field **B**. A binary code is used to represent the state of the assembly, with the first and second digits corresponding to the bottom and top units, respectively. (*B*) Phase diagram showing the magnetic field conditions for the structure to switch from state [00] to states [10], [01], and [11]. Dots are from experimental measurements, fitted by a polynomial function. (*C*) Bidirectional bending of the two-unit Kresling assembly with in-plane magnetizations $M_{i}$ and out-of-plane magnetic field **B**. (*D*) Experimental measurements of the bidirectional bending angle. Dots are from experimental measurements, fitted by sinusoidal functions. (*E* and *F*) Omnidirectional bending of a two-unit Kresling assembly with out-of-plane magnetizations $M_{o}$ and in-plane magnetic field **B** (*E*) and polar plot of the experimental bending angle measurement (*F*). (*G* and *I*) Multimodal deformation of a two-unit Kresling assembly with in-plane (*Top* plate) and out-of-plane (*Bottom* plate at the *Middle* plane) magnetizations and in-plane magnetic field **B**, showing omnidirectional bending at state [00] (*G*) and omnidirectional bending at state [01] (*I*). (*H* and *J*) Experimental measurements of the bending angle polar plot at state [00] (*H*) and polar plot at state [01] (*J*). The gray region in *J* denotes the conditions when the top unit folds.

Although the folded Kresling unit can effectively achieve either deploying or bending, it cannot deploy and bend at the same time. Therefore, we use two-unit Kresling assemblies to show the basic concept of integrated motion that combines Kresling bending with deploying, implemented by the distributed actuation of the magnetic field. In Fig. 2, three two-unit Kresling assemblies with different magnetization combinations are created by attaching two magnetic plates to the top of two Kresling units. Fig. 2*A* shows the first magnetization combination, where both magnetic plates are designed with in-plane magnetizations $M_{i}$ along the same rightward direction at the all-folded state [00]. Note that the binary code represents the state of the assembly, with the first and second digits corresponding to the bottom and top units, respectively. When an in-plane magnetic field **B** is applied with an angle of α to the rightward direction ($M_{i}$ direction at state [00]), in-plane magnetic torques are generated on both magnetic plates, driving the assembly to transform between four stable states via stable folding and deploying (Movie S2 and *SI Appendix*, Fig. S7). From the phase diagram in Fig. 2*B*, starting from stable state [00], stable states [10] and [11] can be directly reached when programming magnetic field intensity *B* and direction α. The stable state [01] can be achieved following stable state [11] (*SI Appendix*, Fig. S8). Bidirectional bending of states [00], [10], and [01] under an out-of-plane magnetic field with an angle of β to the horizontal direction is shown in Fig. 2*C*, with [10] and [01] revealing integrated bending and deploying deformations (Movie S2). Note that the deployed unit cannot bend under the applied magnetic field. Fig. 2*D* shows the experimental measurements of the bending angles (states [00], [10], and [01]) under a 30-mT magnetic field with varying directions (β ranging from −180° to 180°). The bending angle θ is defined as the angle between the horizontal direction and the folded unit’s top plane, showing a sinusoidal relation with respect to β. States [01] and [10] exhibit similar bending behavior with a maximum angle of about 15°, as only one folded unit contributes to the overall bending, and state [00] shows larger bending with a maximum bending angle of about 30°, as the bending is accumulated from two folded units.

The second magnetization combination of the two-unit Kresling assembly has both magnetic plates designed with out-of-plane magnetizations $M_{o}$ (Fig. 2*E*) for omnidirectional bending. Under an applied magnetic field that sweeps in-plane, a homogeneous bending is demonstrated in all directions with a maximum bending angle of 31°, as shown by the polar plot in Fig. 2*F*. The bending angle can be enlarged by increasing the magnetic field intensity *B* or adjusting the angle between $M_{o}$ and **B** (*SI Appendix*, Fig. S9). To integrate omnidirectional bending with folding/deploying deformation in the two-unit Kresling assembly, the third magnetization combination is shown in Fig. 2*G*, with the bottom Kresling unit possessing an out-of-plane magnetization $M_{o}$ for omnidirectional bending and the top Kresling unit possessing an in-plane magnetization $M_{i}$ for folding/deploying (*SI Appendix*, Fig. S10). The omnidirectional bending angles of state [00] (Fig. 2*G*) and state [01] (Fig. 2*I*) under in-plane sweeping magnetic fields are characterized in Fig. 2 *H* and *J*, respectively. Maximum bending angles of 37° (state [00]) and 28° (state [01]) toward the 180° direction are obtained. In both cases, the bending is not homogeneous due to the influence of $M_{i}$, which can be compensated by applying a varying magnetic field. Increasing the magnetic field can lead to larger bending angles, but may also cause folding of the top unit at state [01], denoted by the gray region in Fig. 2*J*.

### Omnidirectional Bending and Deploying of a Four-Unit Kresling Robotic Arm.

**Fig. 3.**
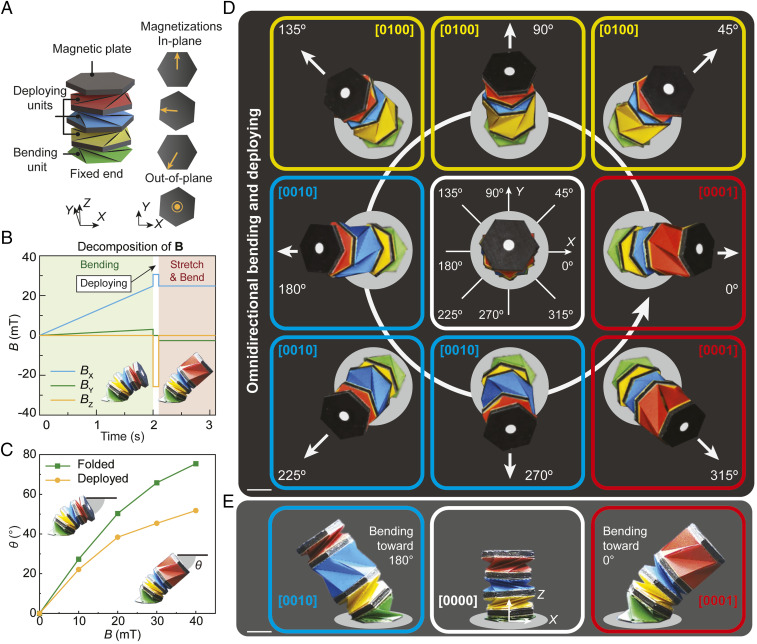
Omnidirectional bending and deploying of a four-unit Kresling robotic arm with relatively large bending angles. (*A*) Schematic design and magnetization distribution of the four-unit robotic arm. The top three units are used for deploying in all directions, and the bottom unit is used for omnidirectional bending. (*B*) An example magnetic field profile (decomposition of 3D magnetic field) of the robotic arm for bending toward the 0° direction and then deploying its top red unit. *B*, *Insets* show the experimental results of the four-unit robotic arm at the folded state (bending only) and deployed state (bending with deploying). (*C*) Experimental bending angle characterization of the robotic arm toward 0° direction without a deployed unit and with the red unit deployed. (*D*) Experimental results from the top view of the robotic arm omnidirectional bending with one deployed unit. Colored contour boxes represent yellow, blue, and red units deployed in the eight directions. A binary code is used to represent the state of the entire robotic arm from the bottom to the top units. (*E*) Experimental results from the front view of the undeformed arm and bending and stretching toward 0° and 180°. (Scale bars: 10 mm.)

Based on the concept of integrated motion with combined omnidirectional bending and deploying of the Kresling units, we next design a Kresling robotic arm consisting of four Kresling units, as shown in Fig. 3*A*, to achieve much larger bending angles with deployability. The green unit with a fixed bottom and an out-of-plane magnetization allows for the capability of omnidirectional bending, and it is called the bending unit (Movie S3). Then, three units (yellow, blue, and red units) with alternating crease directions are added to the axial direction of the robotic arm to provide deployability for the “stretching” behavior of the arm, and they are regarded as the deploying units. Here, the yellow unit and the red unit can be deployed with clockwise torque, and the blue unit can be deployed with counterclockwise torque. The deploying units have in-plane magnetization directions to trigger selective deployment under different magnetic fields (Movie S3). Due to the accumulated deformation from multiple units, the four-unit robotic arm shows large omnidirectional bending and stretching. Fig. 3*B* shows an example magnetic field profile to bend and stretch the structure toward the 0° direction (*X* direction). **B** is a vector field that can be decomposed into three Cartesian directions (${\text{B}}_{\text{X}}$, ${\text{B}}_{\text{Y}}$, and ${\text{B}}_{\text{Z}}$). A 25-mT magnetic field in the *XY* plane is first applied to bend the arm toward the 0° direction. The white marker on the top of the robotic arm illustrates a precisely controlled bending direction toward 0°. Then, an impulse magnetic field (40 mT) parallel to the top magnetic plate is applied to quickly deploy the red unit (*SI Appendix*, *Coordinate Transformations*). To keep the bending direction after stretching, another 25-mT magnetic field that is different from the bending magnetic field is applied as the plate’s magnetization direction changes during the deployment (Fig. 3*B*). Fig. 3*C* characterizes the 0°-direction bending angle θ, defined as the angle between the deformed top plane and the horizontal direction at the robotic arm’s all-folded state (bending only) and deployed state (bending with deploying). With increased magnetic field intensity, the robotic arm shows large bending angles for both states with maximum values 75° (bending only) and 52° (bending with deploying). The bending and stretching of the robotic arm are omnidirectional based on rationally programmed magnetic field profiles (Fig. 3*D* and Movie S4). With the designed deploying units’ crease directions and magnetizations, a specific deploying unit (denoted by the colored contour box) can be deployed when the arm bends to different directions (Fig. 3*E*). Detailed magnetic field profiles to bend and stretch the robotic arms to eight different directions are shown in *SI Appendix*, Fig. S11, and the omnidirectional bending angles at the all-folded state and deployed state are characterized by the polar plots in *SI Appendix*, Fig. S12.

### Octopus-Like Robotic Arm for Stretching, Bending, and Twisting Motions.

**Fig. 4.**
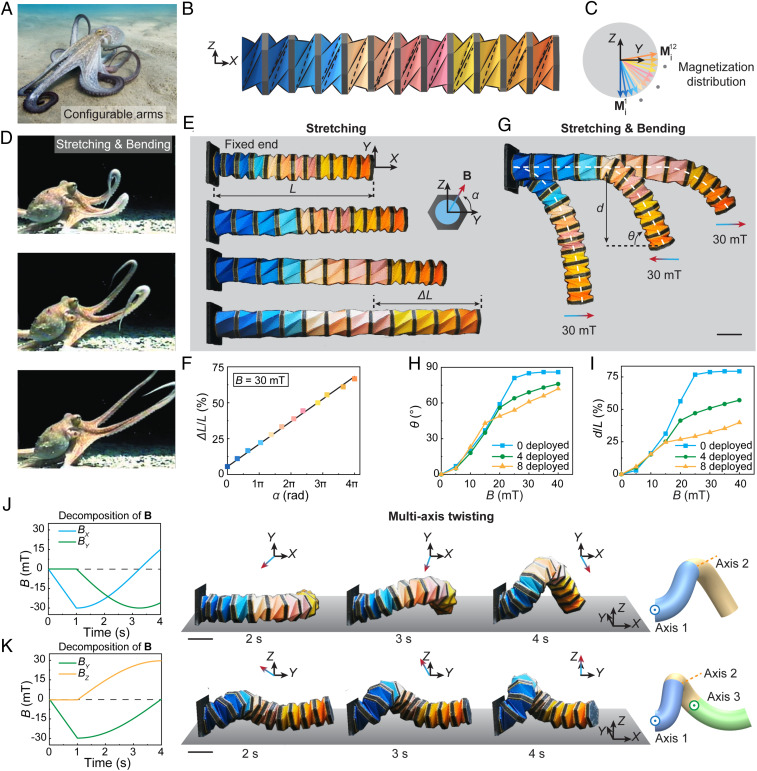
Octopus-like robotic arm with stretching, bending, and twisting motions. (*A*) An octopus with its configurable arms. (*B*) Schematic design of a 12-unit robotic arm for biomimetic motions. (*C*) Magnetization distribution of the robotic arm. (*D*) Photos of stretching and bending behaviors of octopus arms during preying, adapted from ref. 41. (*E*) Experimental results of controlled stretching and contracting of the robotic arm under rotating magnetic fields. (*F*) Stretching ratio Δ*L*/*L* with respect to the direction of the applied rotating magnetic field α in the *YZ* plane. (*G*) Controlled bending behavior with various deployed units. (*H*) Bending angle θ of the robotic arm with respect to the intensity of the applied magnetic field *B* with zero, four, and eight deployed units. (*I*) Normalized deflection *d*/*L* of the robotic arm with respect to the intensity of the applied magnetic field *B* with zero, four, and eight deployed units. (*J*) A rotating magnetic field in the *XY* plane, a twisting motion of the octopus-like robotic arm, and corresponding two bending axes along the arm. (*K*) A rotating magnetic field in the *YZ* plane, a twisting motion of the octopus-arm-like robot, and corresponding three bending axes along the arm. (Scale bars: 20 mm.)

The octopus arms’ configurability to stretch, contract, bend, and twist permits multifunctional motions, such as walking, swimming, and preying (Fig. 4*A*). Inspired by this biosystem, we engineer a 12-unit Kresling robotic arm with all in-plane magnetizations, as shown in Fig. 4*B*. The magnetizations of the magnetic plates are designed to be in the same negative *Z* direction at the all-folded state under compression (*SI Appendix*, Fig. S13). Due to structural resistance of Kresling units and the repulsive forces between the magnetic plates, each unit expands slightly from the flat-folded state, leading to distributed magnetizations, as shown by the side view in Fig. 4*C*. One fascinating feature of octopus arms is the controllable stretching and bending, which allow for a tunable bending point to reach out and interact with the prey, as shown in Fig. 4*D* (41). Our 12-unit robotic arm can achieve controllable deployment with integrated bending to mimic the motion of the octopus arm. Fig. 4*E* illustrates reversible stretching and contracting (Movie S5). During the stretching motion, the units can be deployed sequentially from left to right under a counterclockwise rotating magnetic field parallel to the fixed end (*YZ* plane). The magnetic field direction is defined by a relative angle α to the *Y* direction in the *YZ* plane. Fig. 4*F* illustrates that the stretching ratio Δ*L*/*L* has a linear relationship with the rotation of the magnetic field with a maximum value of 66.7%, where *L* is the initial length of the robotic arm and Δ*L* is the length change. The robotic arm can contract back to the folded state under a clockwise rotating magnetic field. Due to the unevenly distributed magnetizations at the deployed state (*SI Appendix*, Fig. S14), the contraction process of the units is not sequential. However, the arm’s total length can still linearly decrease with respect to time and the rotational magnetic field. Its contracting speed is approximately the same as the stretching speed (*SI Appendix*, Fig. S15).

The robotic arm can also induce bending deformation under a different magnetic field, as shown in Fig. 4*G*. The bending behavior of the robotic arm can be easily coupled with a select number of deployed units (zero units, four units, and eight units in Fig. 4*G* and Movie S5), which resembles the bending and stretching behaviors of the octopus arm (Fig. 4*D*). In this way, the overall length and stiffness of the bent arm is tunable. Fig. 4 *H* and *I* characterize the bending angle θ and normalized deflection (*d*/*L*) of the controllable bending with stretching. The bending angle θ is defined as the angle between the neutral axis at the free end and horizontal direction, and the deflection *d* is defined as the vertical distance between the arm’s two ends. As shown in Fig. 4*H*, with the increasing magnetic field to *B* = 40 mT, θ increases and then reaches 72°, 76°, and 86° for three cases with eight, four, and zero deployed units, respectively. Similarly, the number of deployed unit notably influences the robotic arm’s normalized deflection with a maximum value of 39.8%, 57.1%, and 79.4% for three cases with eight, four, and zero deployed units, as shown in Fig. 4*I*.

More interestingly, 3D out-of-plane shape reconfiguration of the robotic arm can be achieved through integrated bending and twisting motion under programmed magnetic fields. When applying a counterclockwise rotating magnetic field in the *XY* plane starting along the negative *X* direction, the arm first bends in the *XY* plane and then twists out of plane while interacting with the ground (Fig. 4*J* and Movie S5). This bending–twisting is approximately divided into two segments with two different bending axes, as denoted by blue and yellow in the 3D schematic in Fig. 4*J*. By changing the magnetic field to a clockwise rotating magnetic field in the *YZ* plane starting along the negative *Y* direction, the arm first slightly contracts and then buckles out of the *XY* plane with three bending axes, as shown in Fig. 4*K*, leading to a different bending–twisting deformation with three segments (blue, yellow, and green in the 3D schematic). The morphology of twisting can be determined by the time-dependent **B** field.

**Fig. 5.**
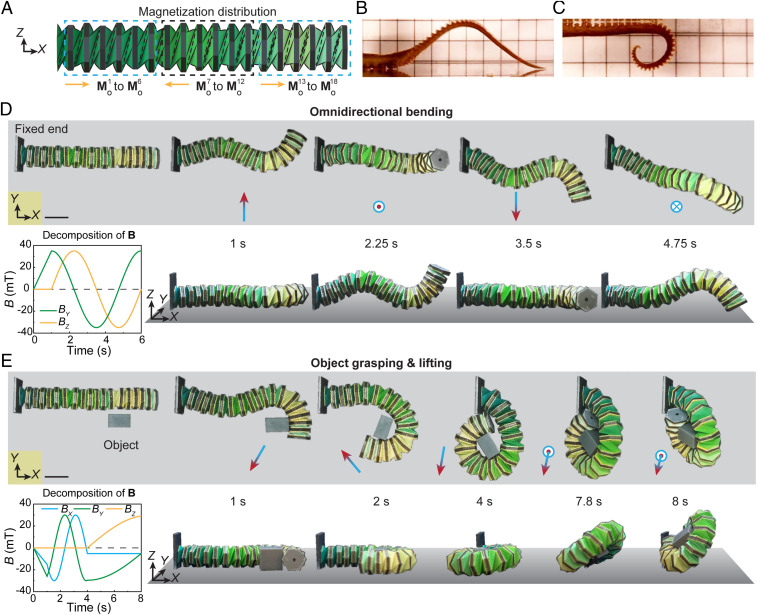
Octopus-like robotic arm with omnidirectional bending and functionality illustrated by object grasping. (*A*) Schematic design and out-of-plane magnetization distribution of an 18-unit robotic arm for biomimetic motions. (*B* and *C*) An octopus wiggles its arms to different curled shapes; adapted from ref. 42. (*D*) Magnetic profile and experimental results of the robotic arm omnidirectional bending motion from top view and front view. (*E*) Magnetic profile and experimental results of the robotic arm object-grasping and lifting motions from top view and front view. (Scale bars: 20 mm.)

Kresling robotic arms have demonstrated the capability of realizing multimodal deformation under well-regulated magnetic control. By designing the magnetization distribution of the multiunit robotic arm, more interesting, highly integrated motions can be achieved. Fig. 5 shows an 18-unit octopus-arm-like robotic arm that can induce omnidirectional bending to interact with objects. The 18 out-of-plane magnetizations are designed with alternating directions for every six units, as illustrated in Fig. 5*A*. An octopus wiggles its arms to different curled shapes to circumvent obstacles, reach out, and get the prey (Fig. 5 *B* and *C*). To mimic the wavy motion in Fig. 5*B* by the robotic arm, a 35-mT magnetic field along the *Y* direction (1 s) is first applied, and the arm morphs into a curled shape in the *XY* plane (Fig. 5*D*). Following by rotating the magnetic field in the *YZ* plane, the 18-unit arm realizes dynamic omnidirectional bending (Movie S6). The curled octopus-arm configuration in Fig. 5*C* can be realized and further explored for functions such as grasping and lifting objects. As demonstrated in Fig. 5*E*, a rotating magnetic field in the *XY* plane is applied to curl the arm tip and grasp an object in 4 s. Then, the applied magnetic field is programmed to rotate about the *X* axis to lift the object in another 4 s (Movie S6).

## Concluding Remarks

We have engineered multifunctional origami robotic arms for biomimetic multimodal deformations and motions with untethered actuation using magnetic fields. By means of synergistically designed Kresling origami assemblies and magnetic controls, several robotic arm designs are demonstrated with integrated deformations of folding, stretching, omnidirectional bending, and twisting. With control of the robotic arm’s agile motions, functional operations, including object grasping and manipulating, become feasible. The magnetic actuation allows untethered and ultrafast on-demand control of the robotic arm and, in the meantime, makes small-scale devices possible (*SI Appendix*, Fig. S16). The omnidirectional bending and integrated motions of demonstrated small-scale robotic arms (*SI Appendix*, Figs. S17 and S18 and Movie S7) can be used to develop miniaturized medical devices for endoscopy, intubation, and catheterization with the functionalities of object manipulation and motion in 3D space. The Kresling robotic arm’s integrated motions, together with the untethered and distributed magnetic actuation, provide an innovative strategy for functional operations, such as navigating, sensing, and interacting with objects in environments with limited access.

## Materials and Methods

Kresling units are fabricated from designed Kresling patterns (*SI Appendix*, Fig. S1) using Tant origami paper (0.14 mm thick) or polypropylene film (0.08 mm thick). Stiff hexagons are attached to the unit’s top and bottom surfaces by using Canson Mi-Teintes paper (0.2 mm thick) or Mylar (0.13 mm). A magnetic plate with designed magnetization is attached to the Kresling unit. Multiple units with specific geometries, materials, and magnetizations are assembled into different robotic arm designs based on applications. More details are provided in *SI Appendix*.

## Supplementary Material

Supplementary File

Supplementary File

Supplementary File

Supplementary File

Supplementary File

Supplementary File

Supplementary File

Supplementary File

## Data Availability

All study data are included in the article and/or supporting information.
