# Supplementary material for: Stretchable origami robotic arm with omnidirectional bending and twisting
Source: Proc Natl Acad Sci U S A. 2021 Aug 30;118(36):e2110023118. doi: 10.1073/pnas.2110023118 (PMC8433528; doi:10.1073/pnas.2110023118)
Supplement: Supplementary File [file pnas.2110023118.sapp.pdf]

PNAS
www.pnas.org

Supplementary Materials for

Stretchable origami robotic arm with omnidirectional bending and twisting

Authors
Shuai Wu[a,1], Qiji Ze[a,1], Jize Dai[a], Nupur Udipi[b], Glaucio H. Paulino[b,2], Ruike Zhao[a,2]

Affiliations
[a] Department of Mechanical and Aerospace Engineering, The Ohio State University, Columbus, OH 43210.
[b] School of Civil and Environmental Engineering, Georgia Institute of Technology, Atlanta, GA 30332.

[1] These authors contributed equally to this work.
[2] Corresponding author. Email: paulino@gatech.edu (G.H.P.), zhao.2885@osu.edu (R.Z.).

The PDF file includes:

Supplementary text
Figures S1-S18
Table S1
Legends for Movies S1 to S7
SI references

Other Supplementary Material for this manuscript includes the following:

Movies S1-S7.

Materials and Methods

1. Sample Fabrication

A. Kresling Pattern. The Kresling units used in the main text have the same geometry but two different sizes as shown in Fig. S1A & B. For the demonstrations of 12-unit and 18-unit robotic arms used in Fig. 4 & 5, the unit size is 75% of the units used in Fig. 1-3. Both units are fabricated using Tant origami paper (0.14 mm thick) with an 80 W CO_2 laser cutter (Orion Motor Tech, China). The small-scale robotic arms use a different Kresling geometry (Fig. S1C) with a comparable cross-section dimension to an endotracheal intubation tube (Fig. S16). The small-scale units are fabricated using polypropylene film (0.08 mm thick) with a mechanical cutter (Cricut Maker, Cricut, Inc., USA). After the patterns are folded, 3M Scotch double-sided tape is used to attach the stiff top and bottom hexagons. Canson Mi-Teintes paper hexagons (0.2 mm thick) are used for the units in the main text and Mylar hexagons (0.13 mm thick) are used for the small-scale units. All fabricated units exhibit bistable properties, and we define the folded state and deployed state as state [0] and state [1], respectively.

B. Magnetic Plates. The magnetic plates are molded by mixing Ecoflex-0030 (Smooth-On, Inc., USA) silicone rubber precursor and magnetic microparticles (NdFeB) (Magnequench, Singapore) with an average size of 100 μm and loadings of 20 vol% or 40 vol%. Hexagonal magnetic plates with a thickness of 2 mm and an edge length of 13 mm or 9.8 mm are used for the units in the main text. Hexagonal magnetic plates with a thickness of 1.0 mm and an edge length of 4.9 mm are used for the small-scale units. After curing at 80 °C for 0.5 h, the magnetic plates are taken out from the molds and magnetized using a homemade magnetizer with a 1.5 T impulse magnetic field. The Kresling units and magnetic plates are assembled using Sil-poxy adhesive (Smooth-On, Inc., USA.) for different demonstrations.

2. Magnetic Properties of Magnetic Plates

The magnetic properties of the magnetic materials are measured using a 7400A vibrating sample magnetometer (Lake Shore Cryotronics, Inc., USA). The magnetic moments of 4 mm×4 mm×1 mm samples are measured. Corresponding remanent magnetic moment densities (M_r) are calculated by dividing the magnetic moment by the sample volume. The M_r of the 20 vol% and the 40 vol% magnetic materials are 112.1 kA m^{-1} and 227.5 kA m^{-1}, respectively.

3. Mechanical Characterizations of the Kresling Unit

A. Folding and Deploying Behaviors. The force-displacement curves of the Kresling unit's folding and deploying processes are measured using a universal testing machine (3344, Instron, Inc., USA). The experimental setup is shown in Fig. S2A and B. Cyclic tension-compression tests are performed to characterize the mechanical properties of fabricated Kresling units as shown in Fig. S2C. The mechanical performance is stable after around 300 cycles. The exhibited hysteresis in the folding-deploying plot comes from energy dissipation, due to the contact and friction between Kresling panels during deploying and folding (1). All fabricated Kresling units are cyclically loaded 300 times for a stable behavior before being used in magnetic actuation in this work. The torque-displacement curve at stable state is derived from the force-displacement curve and displacement-rotation angle relation. See (2) for more details about the measurement and derivation.

B. Bending Behavior. The torque-bending angle curve of the Kresling unit in Fig. 1C is derived from its magnetic bending actuation. As shown in Fig. S3A, a magnetic plate (thickness of 2 mm, edge length of 13 mm, 40 vol% of magnetic particles) with an out-of-plane magnetization $\mathbf{M}_o$ is attached to a Kresling unit. A magnetic field **B** is applied in the horizontal direction, perpendicular to the magnetization of the undeformed magnetic Kresling. The magnetic field's intensity ranges from 0 mT to 40 mT with a 5 mT

interval. The bending angle θ with respect to the magnetic field intensity B is measured, as shown in Fig. S3B. The out-of-plane torque $\mathbf{T}_o$ to bend the unit is derived based on magnetic torque as $\mathbf{T}_o = V(\mathbf{M}_o \times \mathbf{B})$ with a magnitude of:

$$T_o = BM_rV\sin\theta_{BM} = BM_rV\sin(90 - \theta),$$

where M_r is the remanent magnetic moment density 227.5 kA m^{-1}, V is the volume of the magnetic plate 0.88 cm^3, θ_{BM} is the angle between magnetic field and the magnetization direction, θ is the unit's bending angle.

4. Magnetic Actuation Setup

All demonstrations are performed using a 3D Helmholtz coil system shown in Fig. S4. Three pairs of standard Helmholtz coils are configured orthogonally to each other. The coils can generate 2.96 mT A^{-1}, 2.97 mT A^{-1}, and 2.90 mT A^{-1} uniform magnetic fields within a space of 160 mm by 120 mm by 80 mm (X-axis, Y-axis, and Z-axis), respectively. The magnetic field direction and intensity can be manipulated by controlling the currents in the three pairs of coils.

5. Coordinate Transformations

For the magnetic actuation of the four-unit robotic arm, both global and local coordinate systems are used to realize bending and deploying. The global XYZ coordinate system is based on the 3D coils and the local xyz coordinate system is fixed at the top unit. To generate the magnetic field with specific direction and intensity in the 3D space, the magnetic field vector $\mathbf{B}$ can be decomposed to three axes of the global coordinate system and expressed as:

$$\mathbf{B} = B_X\mathbf{X} + B_Y\mathbf{Y} + B_Z\mathbf{Z},$$

where B_X, B_Y, and B_Z are the magnetic fields generated by the pair of coils in the *X*-axis, *Y*-axis, and *Z*-axis, respectively. **B** can also be decomposed to three axes of the local coordinate system and expressed as:

$$\mathbf{B} = B_x\mathbf{x} + B_y\mathbf{y} + B_z\mathbf{z},$$

where B_x, B_y, and B_z are the magnetic fields in the *x*-axis, *y*-axis, and *z*-axis of the local *xyz* coordinate system, respectively. Considering the magnetic field in 3D space is uniform, the transformation between the global *XYZ* and local *xyz* coordinate systems requires only rotation and can thus be expressed as:

$$\begin{bmatrix} B_X \\ B_Y \\ B_Z \end{bmatrix} = \mathbf{Q} \begin{bmatrix} B_x \\ B_y \\ B_z \end{bmatrix},$$

where **Q** is the transformation matrix between two coordinate systems. The designed four-unit robotic arm can bend toward any direction (denoted by γ) in the *XY*-plane with a bending angle of θ (Fig. S11). The transformation matrix **Q** can be expressed as:

$$\mathbf{Q} = \begin{bmatrix} \cos\theta\cos^2\gamma + \sin^2\gamma & \cos\theta\cos\gamma\sin\gamma - \cos\gamma\sin\gamma & \sin\theta\cos\gamma \\ \cos\theta\cos\gamma\sin\gamma - \cos\gamma\sin\gamma & \cos\theta\sin^2\gamma + \cos^2\gamma & \sin\theta\sin\gamma \\ -\sin\theta\cos\gamma & -\sin\theta\sin\gamma & \cos\theta \end{bmatrix}.$$

To realize stretching after the robotic arm's bending deformation, a magnetic field in the *xy*-plane of the local coordinate system should be applied to induce deployment. Then B_X, B_Y, and B_Z can be calculated from the bending deformation (γ and θ) of the robotic arm and the required deploying magnetic field B_x, B_y, and B_z. The reference currents are calculated and sent to the controller of the 3D Helmholtz coils.

6. Magnetic Actuation Experiments

A. Single-unit Kresling Experiments. The bottom plate of the magnetic Kresling unit is fixed and the top plate is free. A magnetic plate (40 vol% of magnetic particles) with an inclined magnetization (60° to

the plate) is attached to the unit. The deploying/folding experiments are conducted by applying a magnetic field in the plane of the fixed end. The direction of the in-plane magnetization component at folded state is defined as the reference direction ($\alpha = 0°$). The intensity of the magnetic field at the direction α increases from 0 mT to 40 mT at 1 mT s^{-1}. The corresponding magnetic fields are recorded when the Kresling unit changes its state from the stable state [0] to the stable state [1] (Fig. 1F) and from the stable state [1] to the stable state [0] (Fig. S5).

The omnidirectional bending behavior is attributed to the out-of-plane magnetization component that can generate out-of-plane torque under the designed magnetic field. To quantitatively evaluate the bending performance, a magnetic field in the plane of the fixed bottom plate is applied with its intensity ranging from 0 mT to 40 mT with 10 mT interval and the direction ranging from 0° to 360° with 30° interval. The relationships between bending angle, magnetic field direction, and magnetic field intensity are illustrated in the polar plot in Fig. 1I.

Experimental actuations of magnetic Kresling units with only in-plane magnetization/out-of-plane magnetization are used to illustrate the actuation mechanisms as shown in Fig. S6. As shown in Fig. S6A, a magnetic plate (40 vol% of magnetic particles) programmed with in-plane magnetization is attached to a blue Kresling unit, which can lead to folding/deploying and bidirectional bending under in-plane and out-of-plane torques, respectively. As shown in Fig. S6B, a magnetic plate (40 vol% of magnetic particles) programmed with out-of-plane magnetization is attached to a green Kresling unit, which enables omnidirectional bending induced by the out-of-plane torques in all directions.

B. Two-unit Kresling Experiments. In Fig. 2, three two-unit Kresling assemblies with different magnetization distributions are explored to demonstrate the deformations including stable folding/deploying and bidirectional bending (blue-blue assembly), omnidirectional bending (green-green

assembly), and multimodal deformation (green-blue assembly). For all demonstrations, the bottom of the Kresling assembly is fixed and the top is free.

For the demonstration of stable folding/deploying and bidirectional bending (blue-blue assembly), two magnetic plates with 20 vol% of magnetic particles and the same in-plane magnetization are attached to the Kresling units as shown in Fig. S7A. The magnetization direction at state [00] is defined as the reference direction ($\alpha = 0°$). The measured phase diagrams of state shifting from states [00], [10], [11], and [01] to other states are shown in Fig. S8. The bidirectional bending deformations of the two-unit Kresling assembly at three states [00], [10], and [01] are demonstrated by applying out-of-plane magnetic fields as shown in Fig. S7B. The characterizations of the bending behaviors at three states are measured by applying a 30 mT magnetic field with an angle of β relative to the magnetization direction at the undeformed state.

For the demonstration of omnidirectional bending (green-green assembly), two magnetic plates (20 vol% of magnetic particles) with out-of-plane magnetizations are attached to the assembly as shown in Fig. S9A. A magnetic field in the plane of the fixed end is applied with its intensity ranging from 0 mT to 40 mT with 10 mT interval and the direction ranging from 0° to 360° with 30° interval. The characterization of bending angle with respect to magnetic field directions and intensities are shown by the polar plot in Fig. S9B.

For the demonstration of multimodal deformation in Fig. S10A and S10C (green-blue assembly), a magnetic plate (40 vol% of magnetic particles) with an out-of-plane magnetization is attached to the green unit to provide omnidirectional bending, and a magnetic plate (20 vol% of magnetic particles) with an in-plane magnetization is attached to the blue unit to provide state shifting. The same in-plane magnetic fields are applied for the characterizations of bending behaviors at state [00] and state [01] (Fig. S10B and S10D).

C. Four-unit Kresling Robotic Arm. The actuation of the four-unit Kresling robotic arm has a bending phase and a deploying phase as shown in Fig. S11. The white marker on the top plate is used to indicate the bending direction of the robotic arm (0° to 360° with 45° interval) and to evaluate whether it follows the desired actuation direction.

The bending angles of the four-unit Kresling robotic arm at 8 different directions (0° to 360° with 45° interval) are measured. At each direction, the magnetic field is applied with an intensity ranging from 0 mT to 40 mT with 10 mT interval. The experimental results are illustrated by the polar plots in Fig. S12.

D. 12-unit Kresling Robotic Arm. In the experiment, the left-end of the 12-unit Kresling robotic arm is fixed, and the rest of the robotic arm is free to generate stretching/contracting, bending, and twisting motions under rationally designed magnetic fields. All the magnetic plates (20 vol% of magnetic particles) are programmed to be in the same negative Z-direction at the all-folded state as shown in Fig. S13. By applying a rotating magnetic field in the YZ-plane as shown in Fig. S15A, all 12 units can be deployed one-by-one from the folded state. By applying a rotating magnetic field in the YZ-plane as shown in Fig. S15B, the arm can gradually decrease its length and return to all folded state.

E. 18-unit Kresling Robotic Arm. The left-end of the 18-unit Kresling robotic arm is fixed, and the rest of the robotic arm is free to generate omnidirectional bending and object grasping motions with rationally designed magnetic fields. All magnetic plates (20 vol% of magnetic particles) in the 18-unit Kresling robotic arm are programmed with out-of-plane magnetizations. The magnetization distribution is shown in Fig. 5A.

F. Small-scale Kresling Robotic Arms. The eight-unit and four-unit small-scale Kresling robotic arms are hanging straight down with the top-end fixed. Both arms provide versatile motion including bending, folding and weight manipulating under programmable magnetic fields. For the eight-unit arm, all magnetic plates (20 vol% of magnetic particles) are programmed with out-of-plane magnetizations, demonstrating bend-lifting and three-dimensional motion of a 1 g weight (Fig. S17, Movie S7). For the four-unit small-scale robotic arm, all magnetic plates (40 vol% of magnetic particles) are programmed with in-plane magnetizations, demonstrating both fold-lifting and bend-lifting of a 1 g weight (Fig. S18, Movie S7)

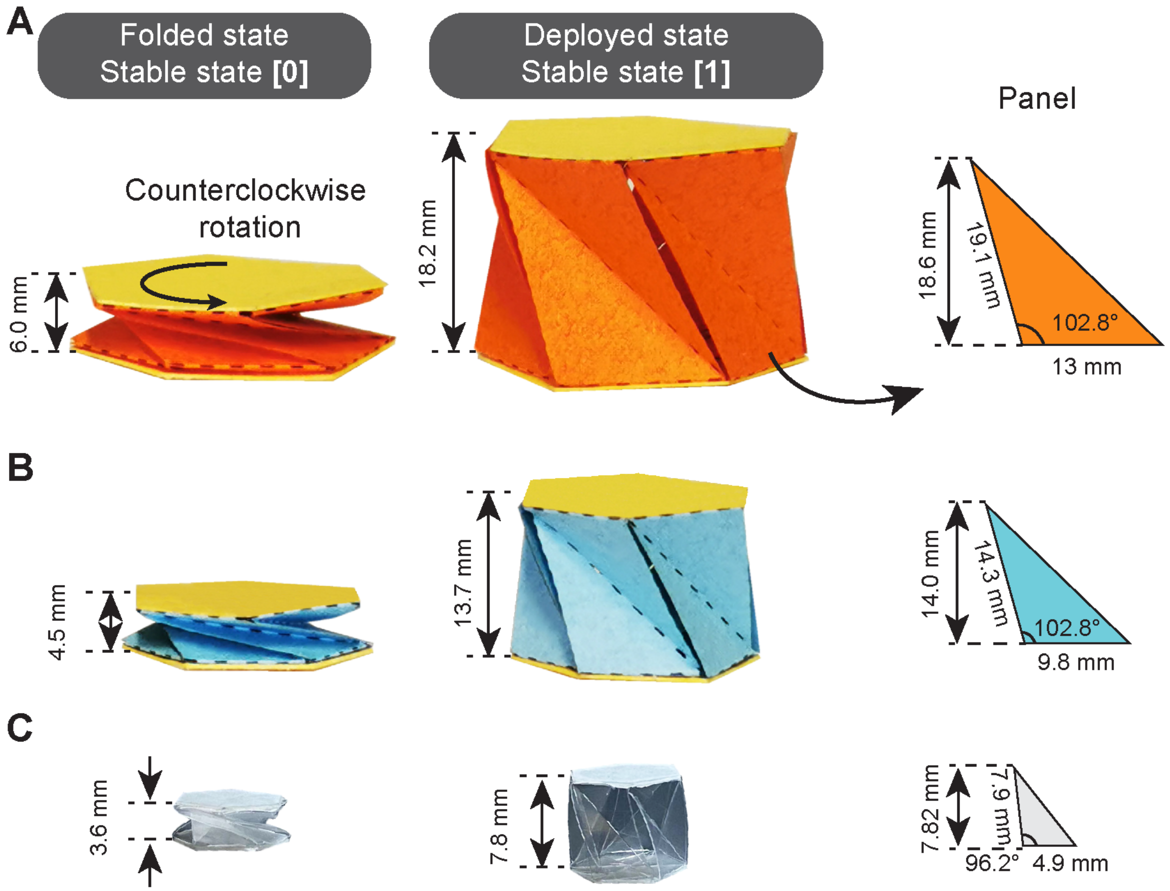

Fig. S1. Images and dimensions of the Kresling units used in this work. Unit pictured in (A) is designed for demonstrations of the single-unit (Fig. 1), two-unit assemblies (Fig. 2), and four-unit robotic arm (Fig. 3). Unit pictured in (B) is designed for arms including the 12-unit (Fig. 4) and 18-unit (Fig. 5) octopus-like robotic arms. Unit pictured in (C) is designed for the small-scale arms (Fig. S16-S18).

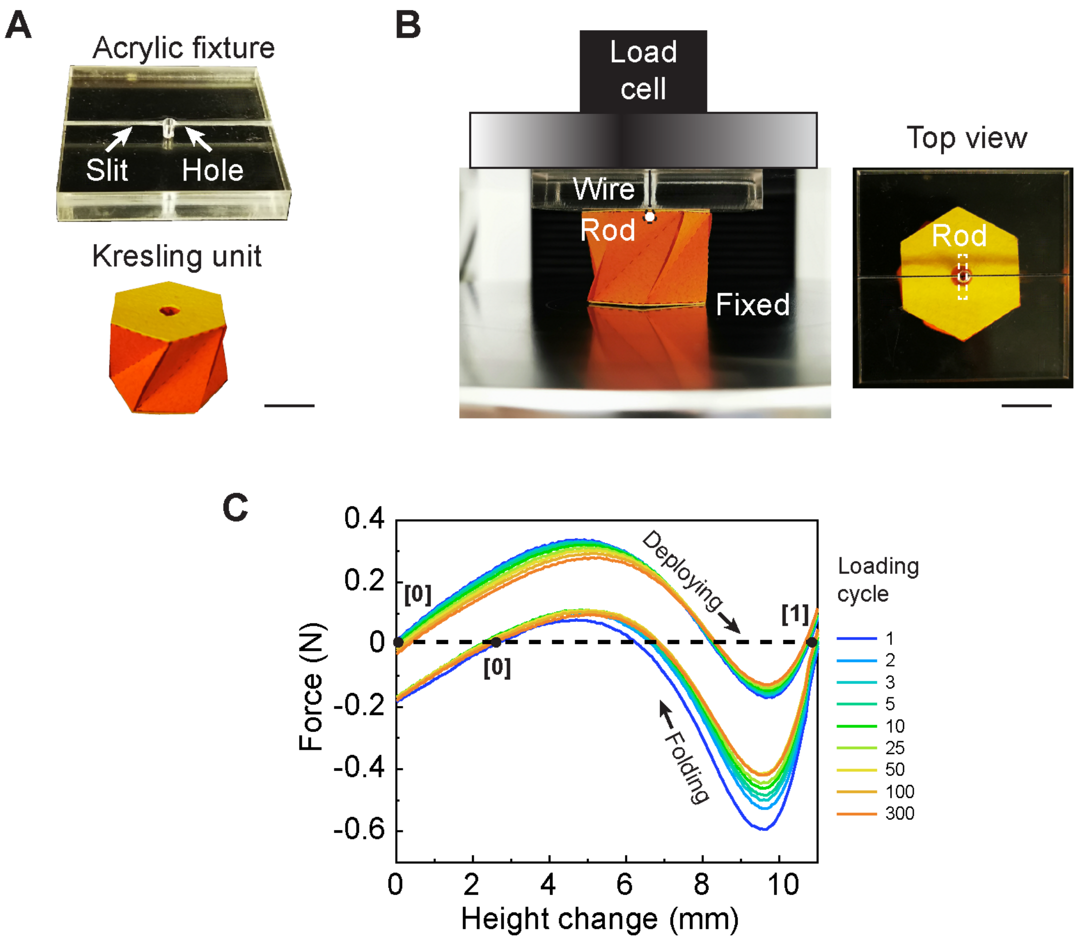

Fig. S2. Mechanical characterization of a Kresling unit. (A) Acrylic fixture and Kresling unit for deploying-folding test. (B) Experimental setup of the tension and compression tests. A wire is used to connect the unit and the rod in the unit to constrain displacement without influencing rotation. (C) The mechanical response of fabricated Kresling unit under cyclic deploying-folding tests. Scale bars: 10 mm.

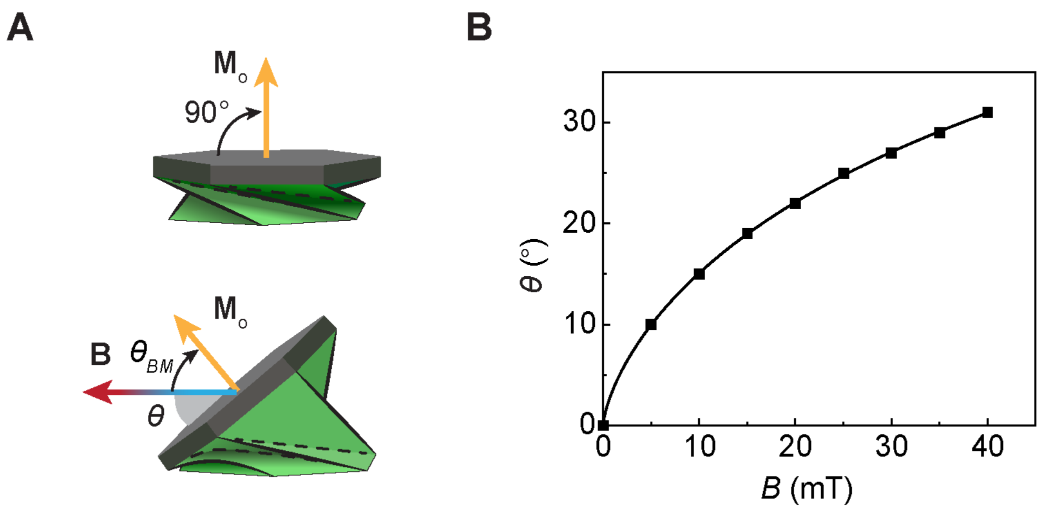

Fig. S3. Experimental measurement of the magnetically actuated bending behavior of the Kresling unit. (A) An out-of-plane magnetized plate is attached to the unit. (B) Bending angle versus magnetic field. Dots are from experimental measurement, fitted by a polynomial function.

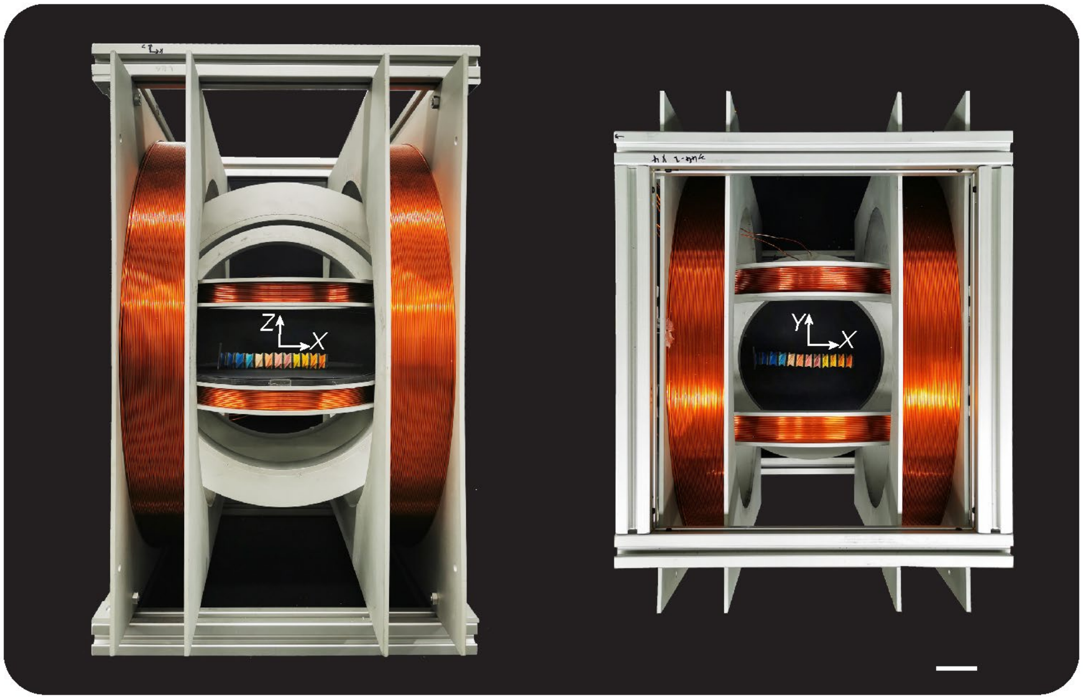

Fig. S4. Front view and top view of the 3D Helmholtz coils with a 12-unit octopus-like robotic arm inside the magnetic apparatus. Scale bar: 5 cm.

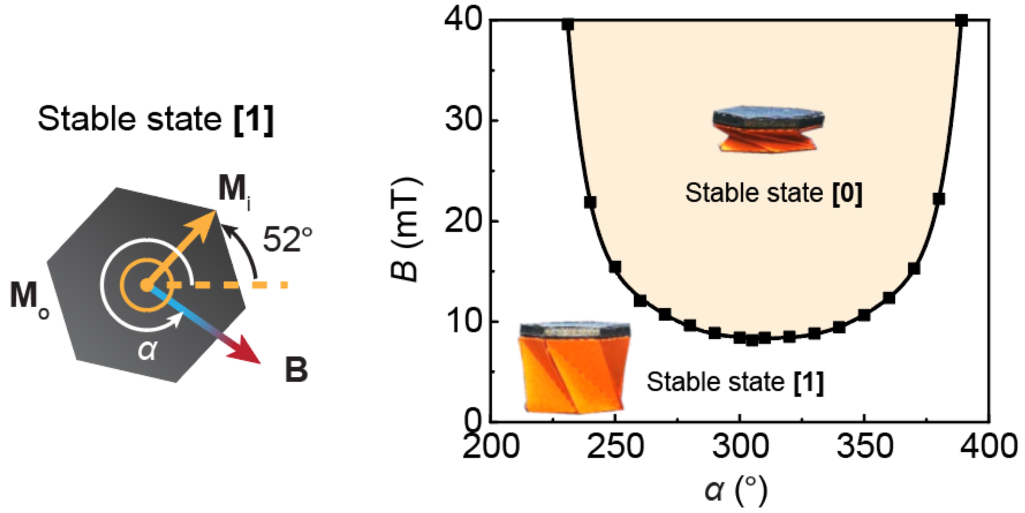

Fig. S5. Experimental measurement of the contour plot shows the magnetic field conditions for the magnetic Kresling to switch from the stable state [1] to the stable state [0]. The dashed orange line represents the in-plane magnetization component direction at the state [0] while the solid orange arrow rotated by 52° corresponds to the magnetization direction at state [1]. Dots are from experiment measurements, fitted by a polynomial function.

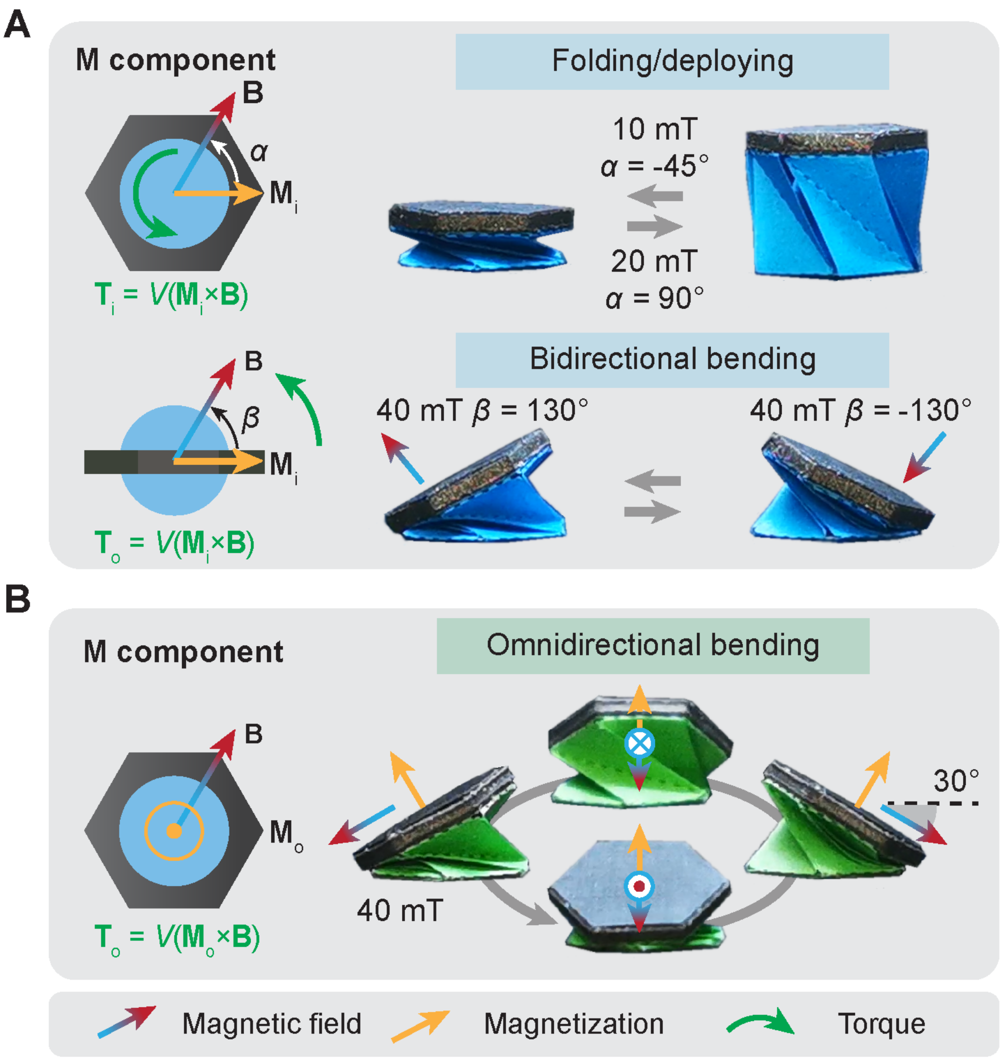

Fig. S6. Experimental results of *folding/deploying* and *bending* of Kresling units (A) *Folding/deploying* and *bidirectional bending* induced by an in-plane magnetization component $\mathbf{M}_i$. (B) *Omnidirectional bending* induced by an out-of-plane magnetization component $\mathbf{M}_o$. A Magnetic field inclined by 30° can help achieve a larger omnidirectional bending angle in (B).

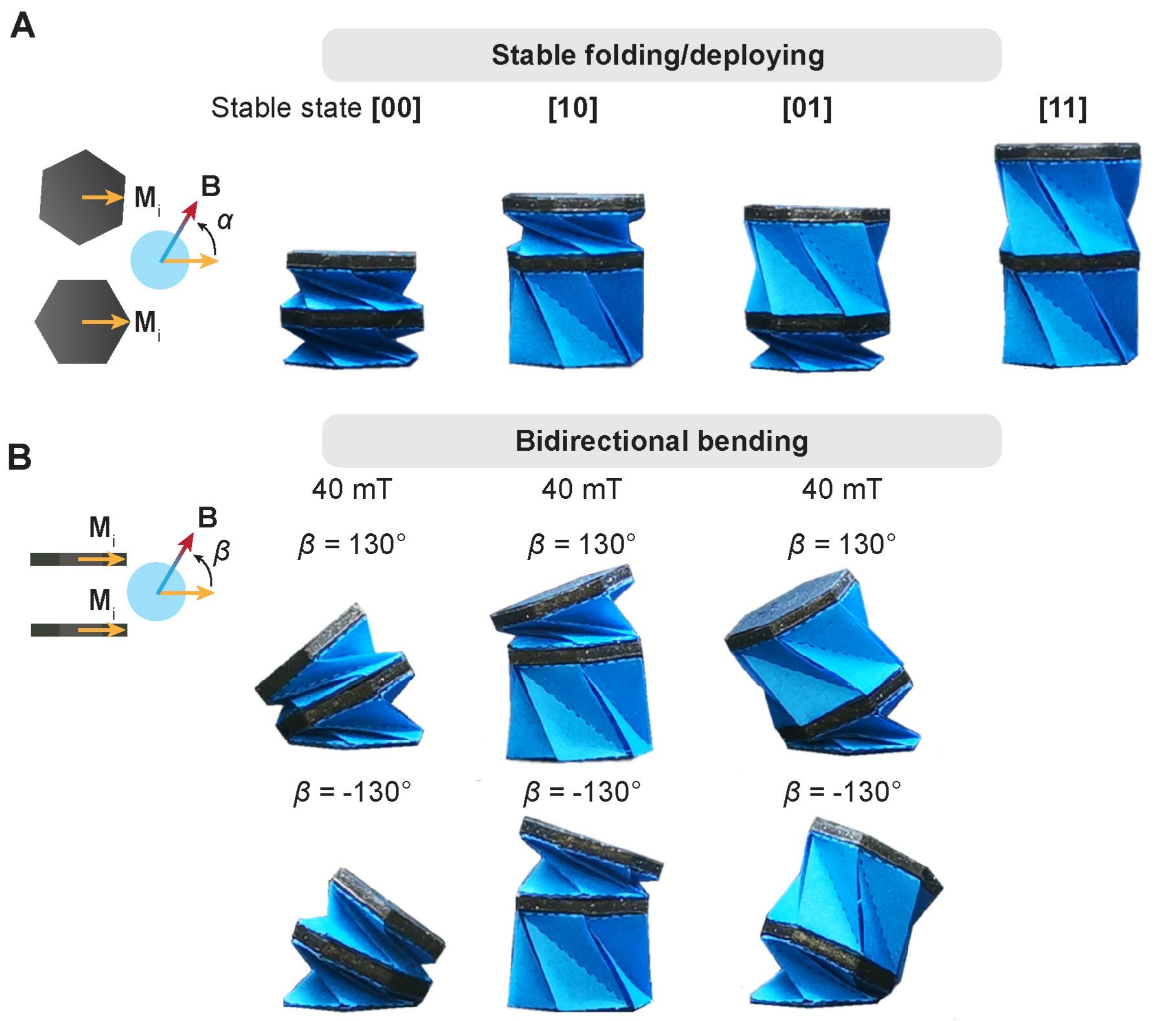

Fig. S7. Experimental results of *folding/deploying* and *bidirectional bending* of two-unit magnetic Kresling assembly with in-plane magnetizations. (A) *State shifting* of the two-unit Kresling assembly with in-plane magnetic fields. (B) *Bidirectional bending* of the two-unit Kresling assembly under the applied out-of-plane magnetic fields.

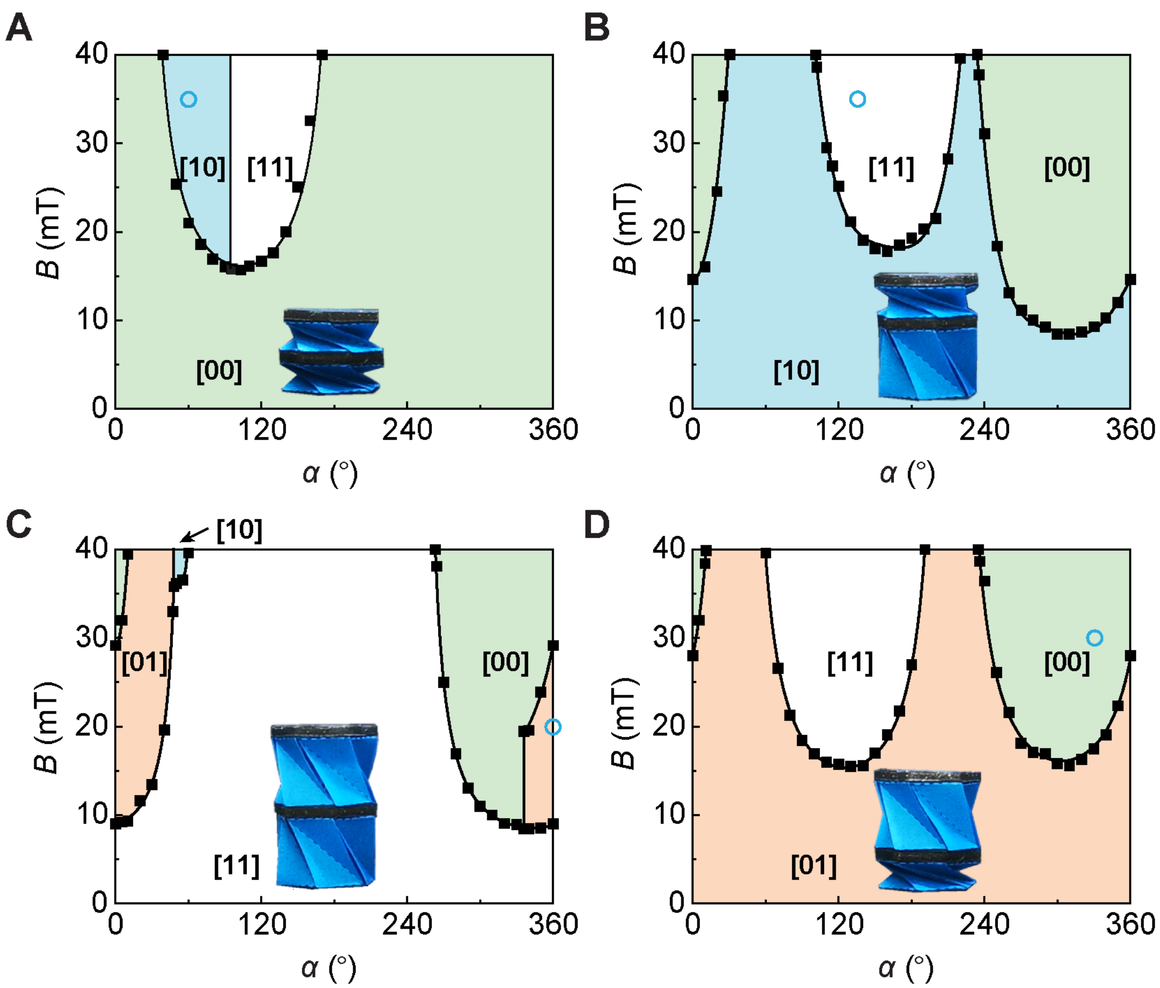

Fig. S8. Experimental results of state shifting phase diagram of the two-unit Kresling assembly with in-plane magnetizations. Actuation results from (A) state [00], (B) state [10], (C) state [11], and (D) state [01]. Dots are from experimental measurements, fitted by a polynomial function. Hollow circles in (A-D) correspond to the conditions used in Fig. S7A for a sequential state shifting ([00] to [10] to [11] to [01] to [00]).

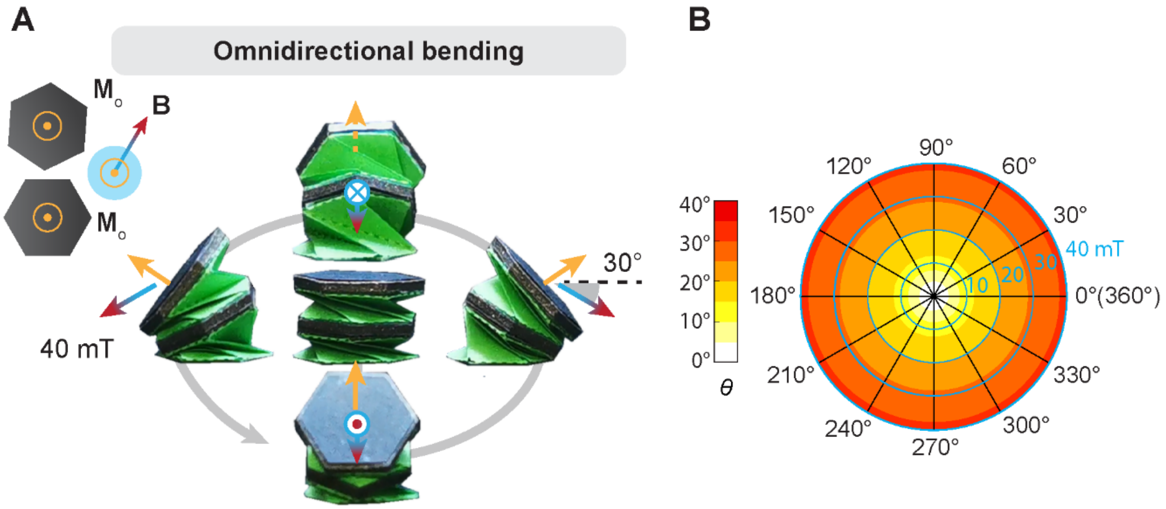

Fig. S9. Experimental results of *omnidirectional bending* (A) and the resulting bending angle polar plot (B) of the two-unit magnetic Kresling assembly with out-of-plane magnetizations. A Magnetic field inclined by 30° can help achieve a larger *omnidirectional bending* angle in (A). A magnetic field in the plane of the fixed end is used for characterizations in (B).

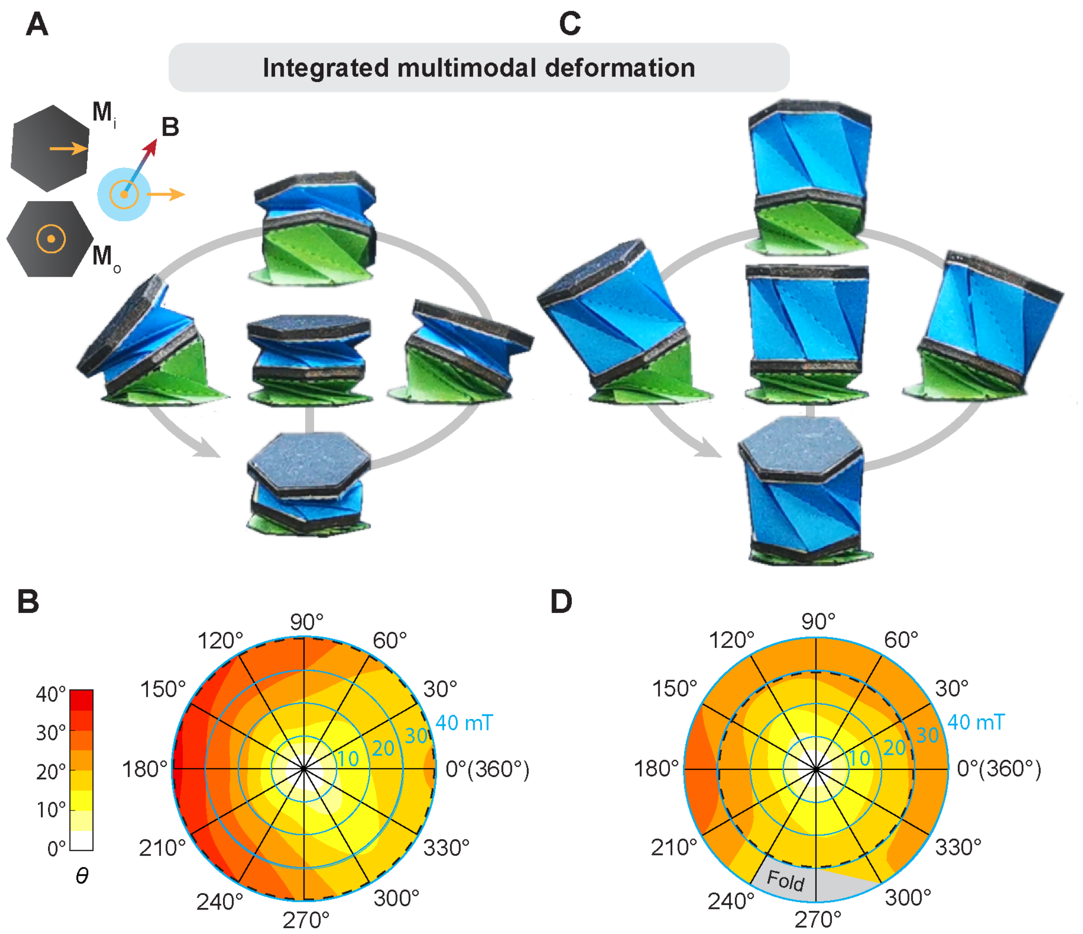

Fig. S10. Experimental results of *multimodal actuation* of the two-unit magnetic Kresling assembly with both in-plane and out-of-plane magnetizations. (A-B) Experimental results of *omnidirectional bending* (A) and the resulting bending angle polar plot (B) at state [00]. (C-D) Experimental results of *omnidirectional bending* (C) and the resulting bending angle polar plot (D) at state [01]. The grey area in (D) denotes the conditions when the top unit folds. Magnetic fields in the plane of the fixed end are used for characterizations in (B) and (D). The dashed lines in (B) and (D) correspond to conditions used in (A) and (C).

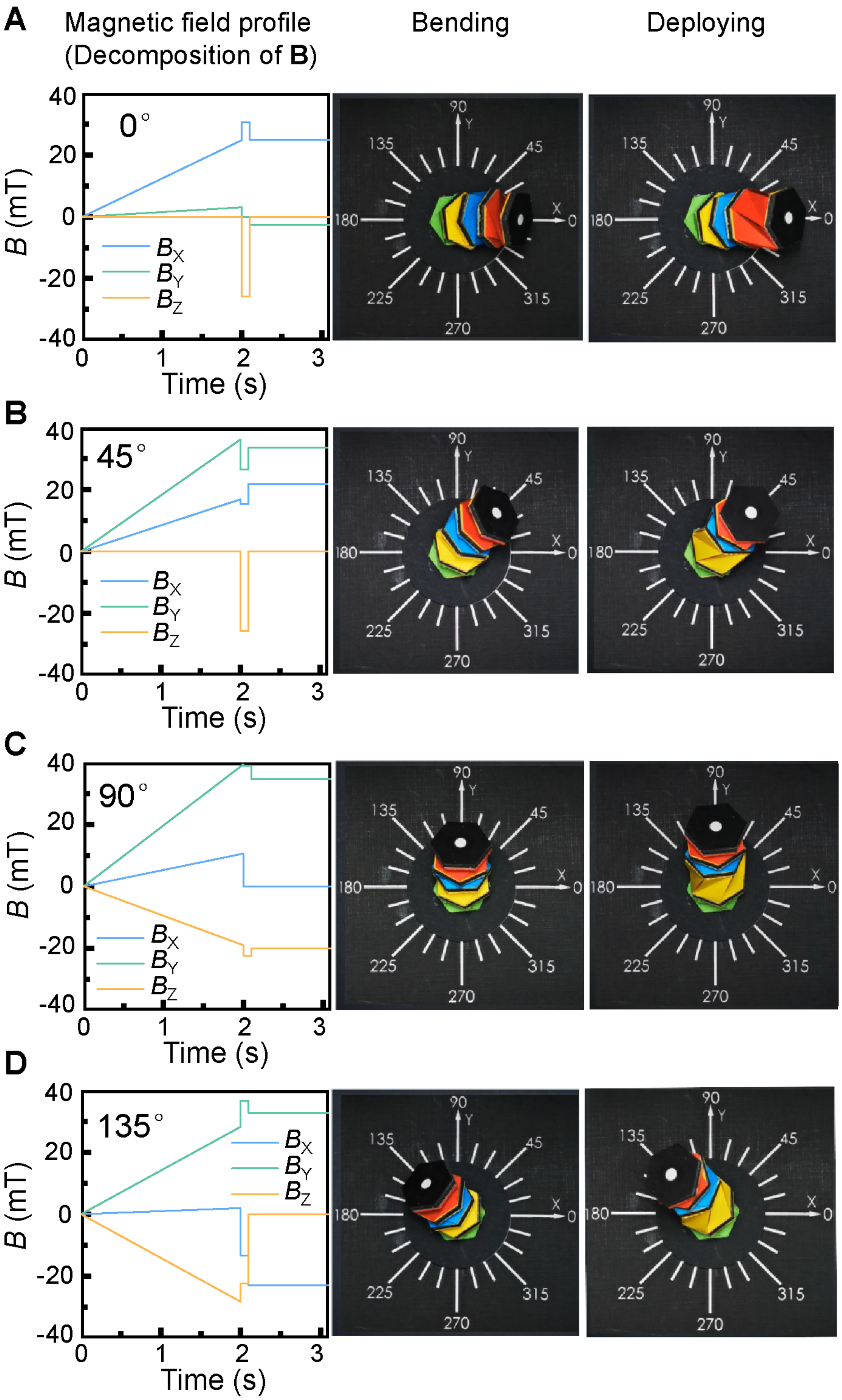

A
Magnetic field profile
(Decomposition of B)
Bending
Deploying
0°
45°
90°
135°
B (mT)
Time (s)
B_X
B_Y
B_Z
B
C
D

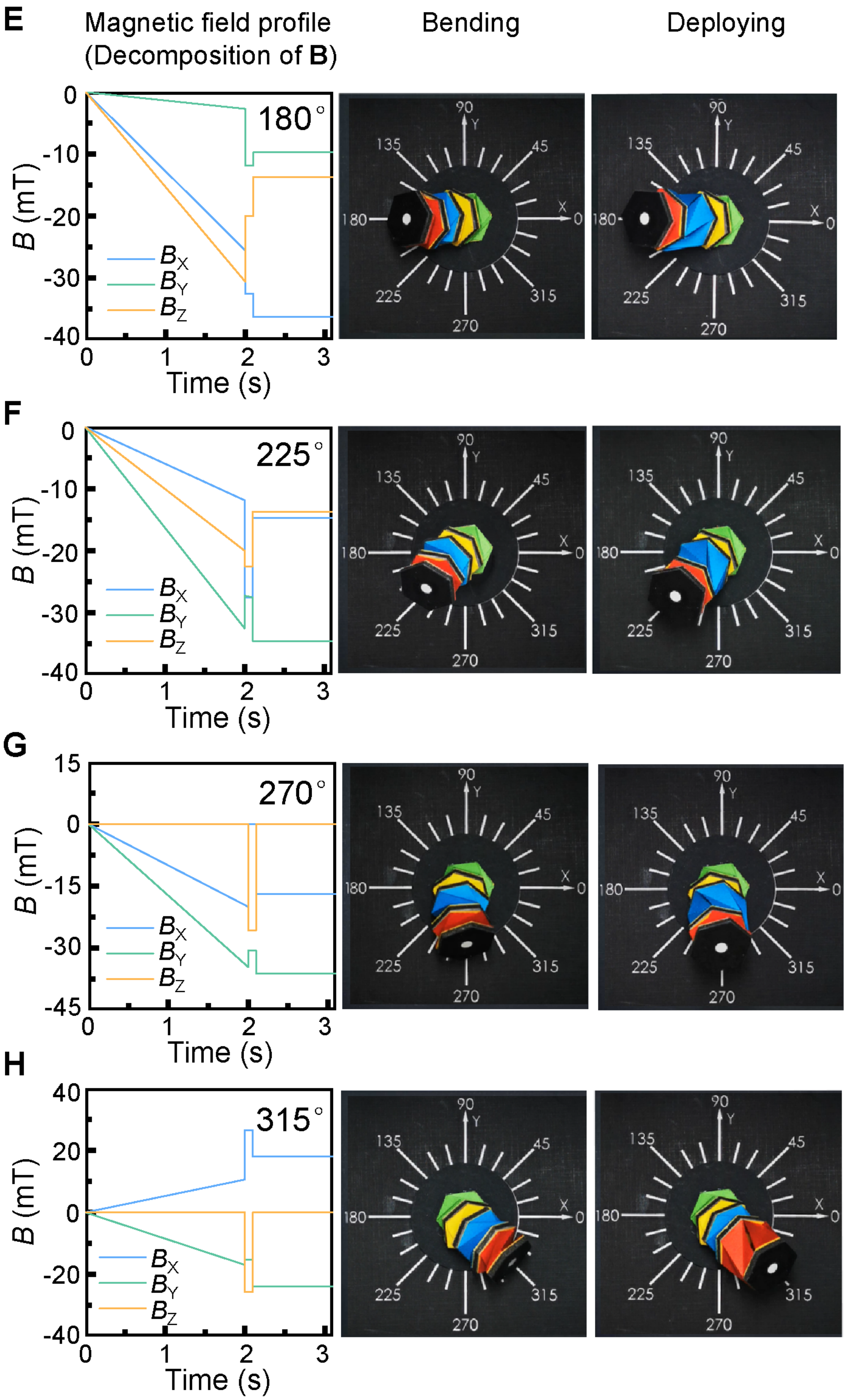

Fig. S11. Magnetic field profiles of four-unit robotic arm bending and deploying at (A) 0°, (B) 45°, (C) 90°, (D) 135°, (E) 180°, (F) 225°, (G) 270°, and (H) 315° directions.

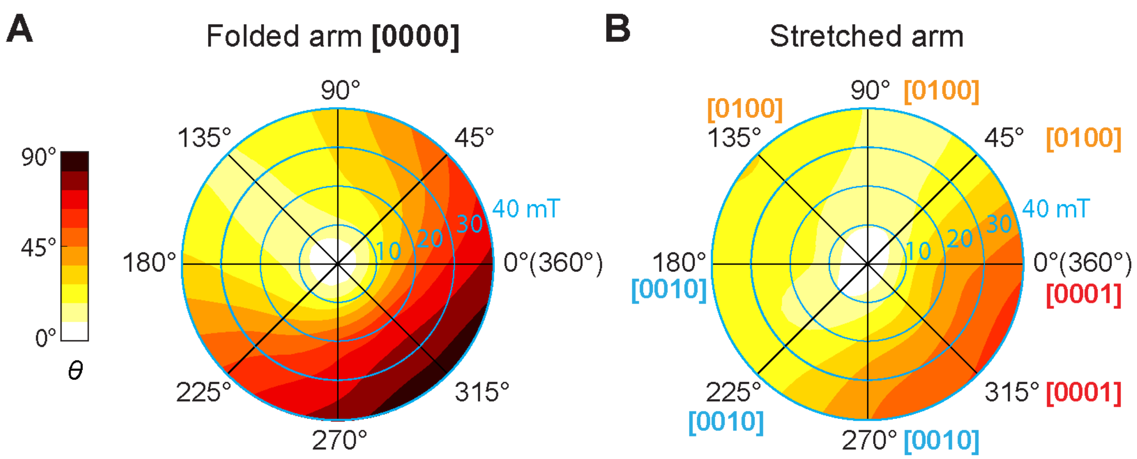

Fig. S12. Bending angle polar plots of the (A) folded arm and (B) stretched arm of the four-unit robotic arm.

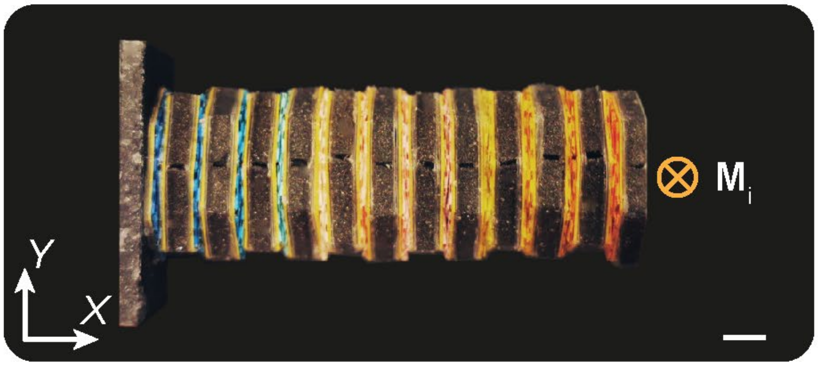

Fig. S13. The magnetizations of the 12-unit robotic arm are programmed to be in the same negative Z-direction at the all-folded state under compression.

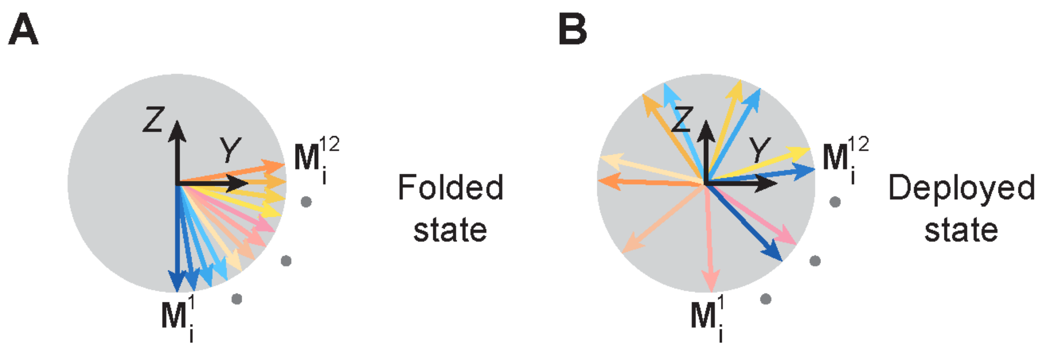

Fig. S14. Magnetization distributions of the 12-unit robotic arm at folded state and deployed state.

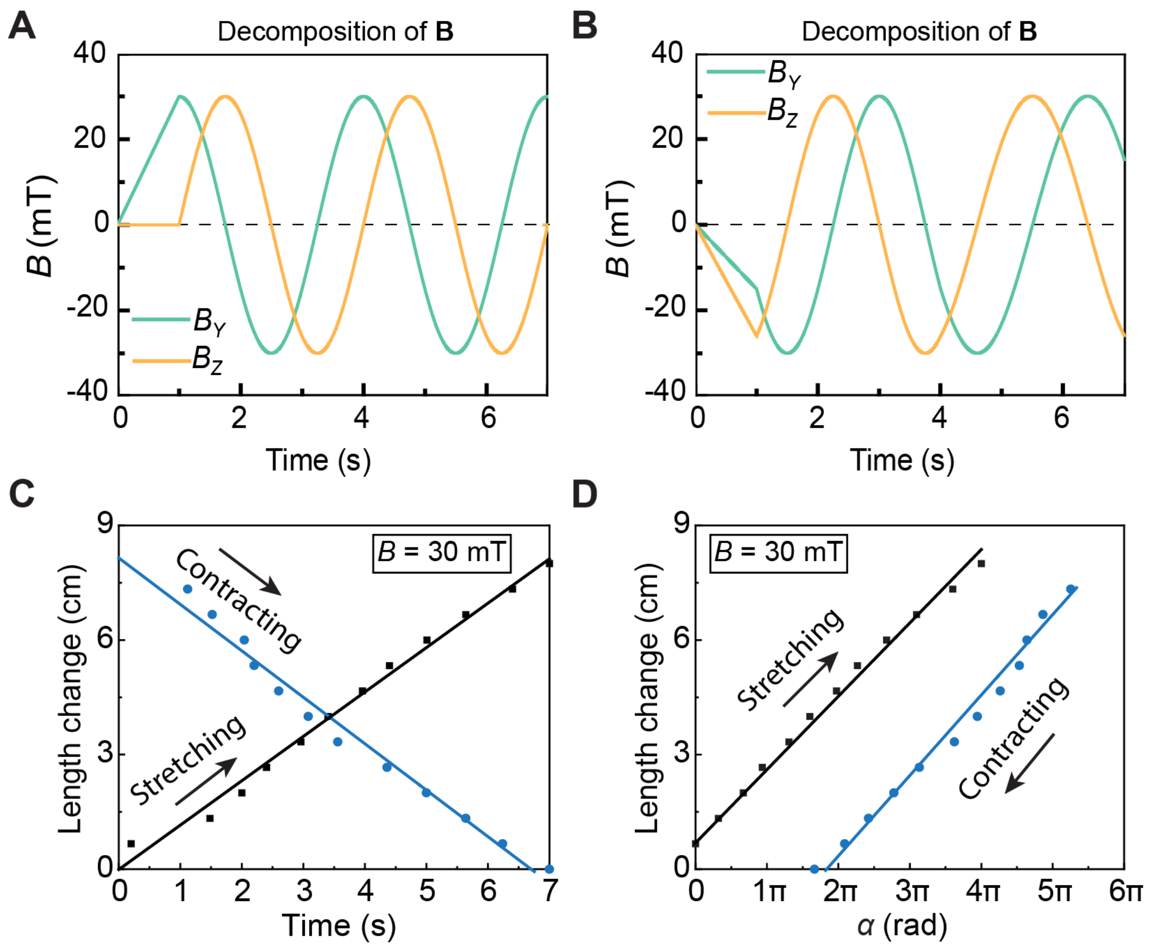

Fig. S15. Control of Kresling robotic arm's stretching and contracting motions. (A) Magnetic profile for stretching motion. (B) Magnetic profile for contracting motion. (C) Length change of the robotic arm during stretching and contracting with respect to time. (D) Length change of the robotic arm during stretching and contracting with respect to the magnetic field direction.

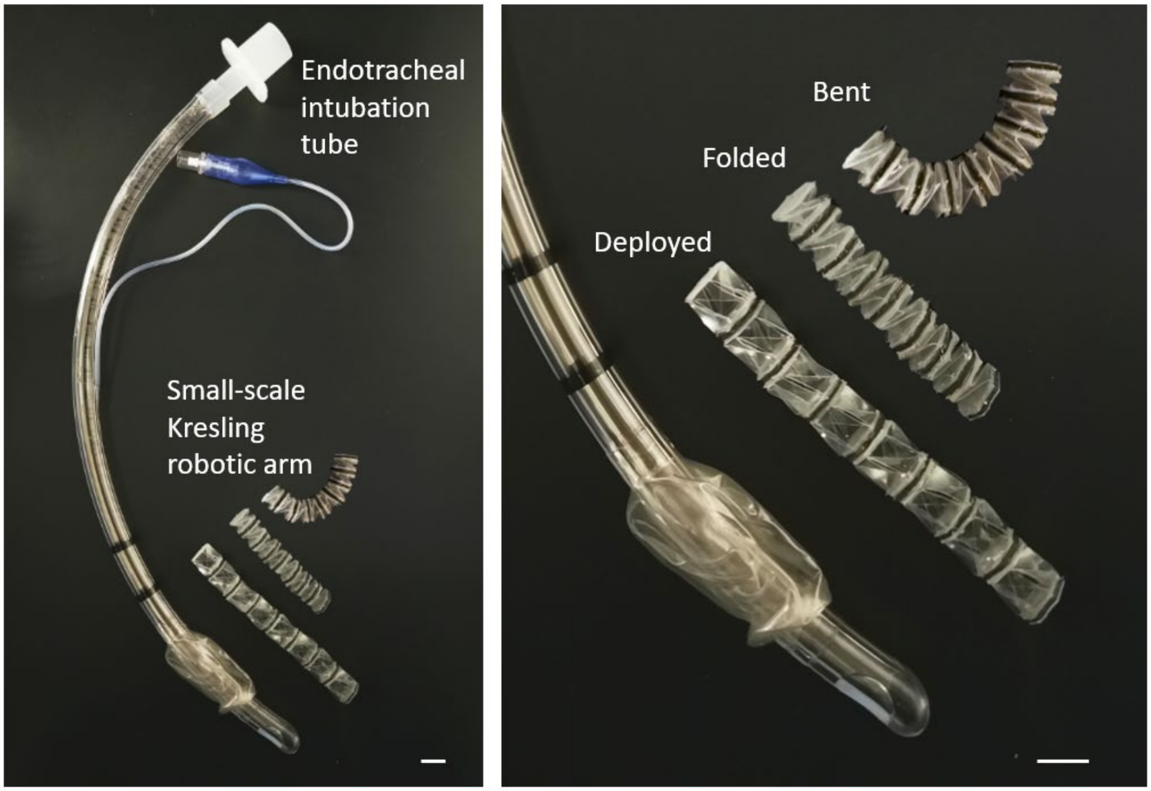

Fig. S16. Prototype of a small-scale eight-unit robotic arm with a comparable cross-section dimension to an endotracheal intubation tube. The aim of this design is to provide well-controlled motion at the tip of medical tubes and catheters to guide the difficult navigation and positioning process during intubation (3), upper endoscopy (4), or catheterization (5) and to achieve object manipulation capability. Scale bars: 10 mm.

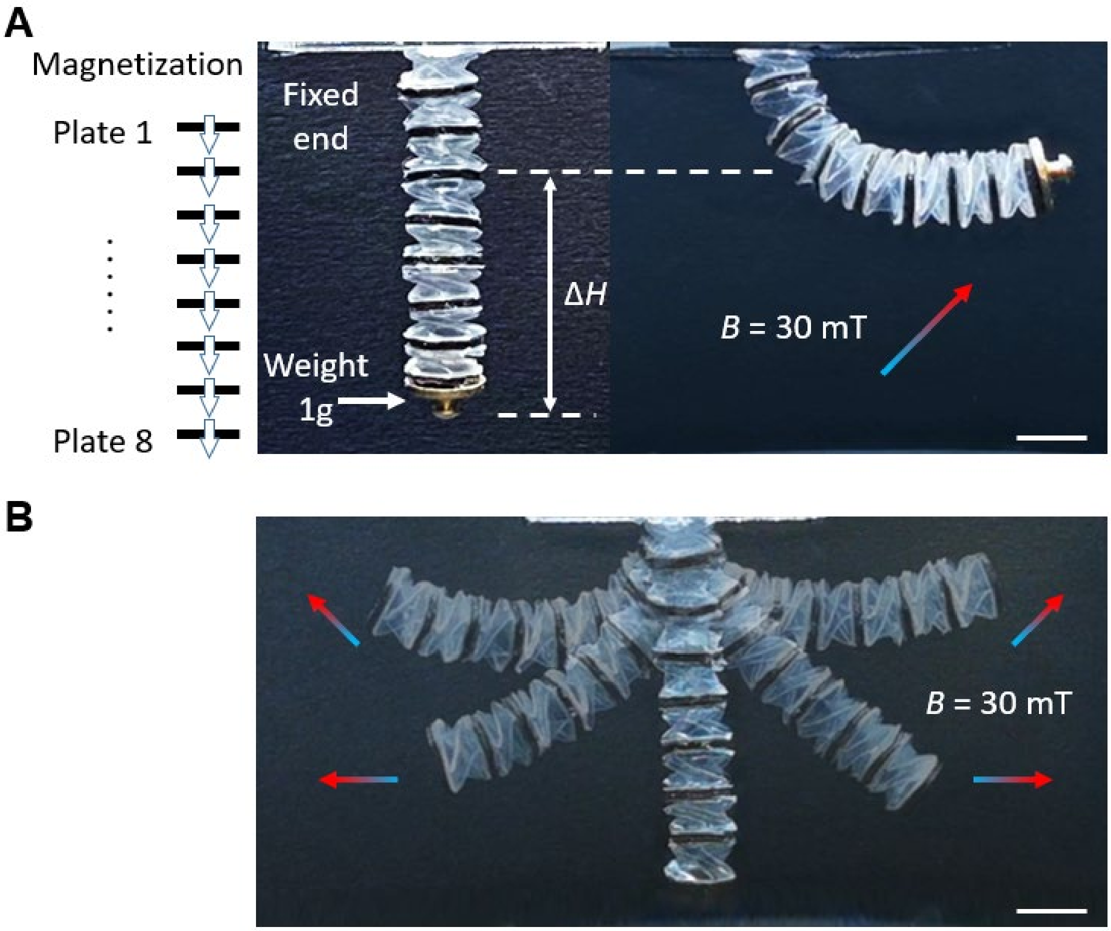

Fig. S17. (A) Bend-lifting and weight motion of the small-scale eight-unit Kresling robotic arm. (B) Versatile motion of the robotic arm under programmable magnetic fields. Scale bars: 10 mm.

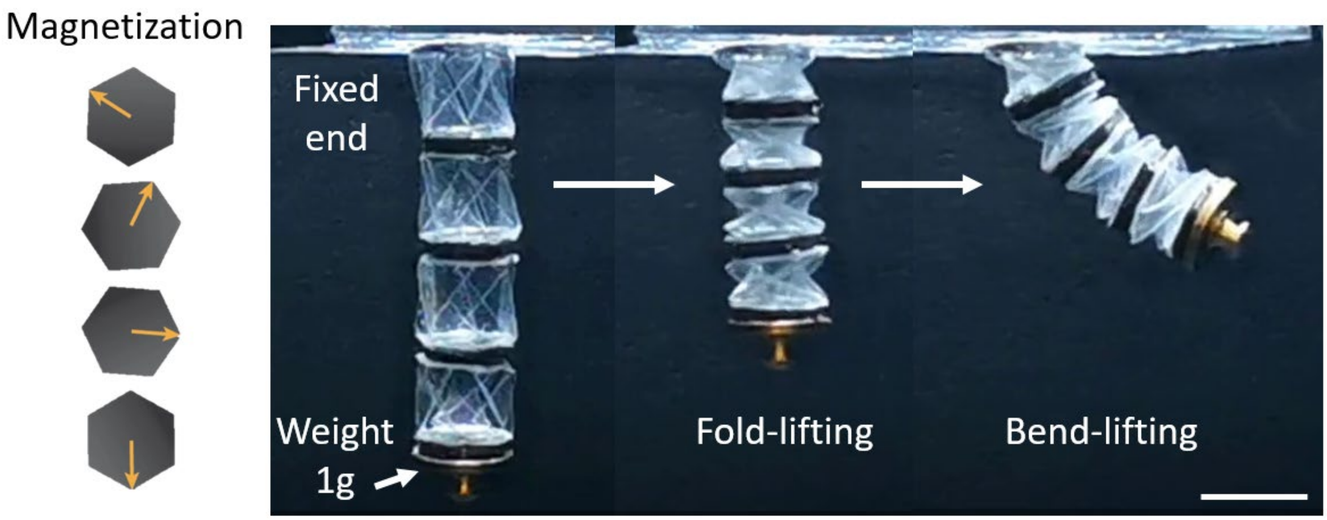

Fig. S18. Fold-lifting and bend-lifting of the small-scale four-unit Kresling robotic arm. Scale bar: 10 mm.

Table S1. Weights on the robotic arms

Robotic arm design	Particle loading of magnetic plate	Weight of single unit	Total weight of the arm
Four-unit arm (Fig. 3)	Bending unit (40 vol%)	~4.8 g	13.4 g
	Deploying unit (20 vol%)	~2.7 g	
12-unit arm (Fig. 4)	20 vol%	~1.5 g	18.7 g
18-unit arm (Fig. 5)	20 vol%	~1.5 g	28.6 g
Eight-unit small-scale arm (Fig. S17)	20 vol%	~0.18 g	1.8 g
Four-unit small-scale arm (Fig. S18)	40 vol%	~0.33 g	1.5 g

Movie S1. Actuation mechanisms of the robotic Kresling unit

Movie S2. Actuation mechanisms of the two-unit Kresling assemblies

Movie S3. Actuation mechanisms of the four-unit robotic arm

Movie S4. Omnidirectional actuation of the four-unit robotic arm

Movie S5. Octopus-like robotic arm: stretching, bending & twisting motions

Movie S6. Octopus-like robotic arm: omnidirectional bending & object grasping

Movie S7. Small-scale robotic arm for biomedical environment

Reference

1. Nayakanti, N., Tawfick, S. H., & Hart, A. J. (2018). Twist-coupled kirigami cells and mechanisms. *Extreme Mechanics Letters*, *21*, 17-24.
2. Novelino, L. S., Ze, Q., Wu, S., Paulino, G. H., & Zhao, R. (2020). Untethered control of functional origami microrobots with distributed actuation. *Proceedings of the National Academy of Sciences*, *117*(39), 24096-24101.
3. Walrath, B. D., Harper, S., Barnard, E., Tobin, J. M., Drew, B., Cunningham, C., ... & Martin, M. (2018). Airway management for trauma patients. *Military medicine*, *183*(suppl_2), 29-31.
4. Ochiai, Y., Kato, M., Kiguchi, Y., Akimoto, T., Nakayama, A., Sasaki, M., ... & Yahagi, N. (2019). Current status and challenges of endoscopic treatments for duodenal tumors. *Digestion*, *99*(1), 21-26.
5. Forsberg, A., & Engström, Å. (2018). Critical care nurses' experiences of performing successful peripheral intravenous catheterization in difficult situations. *Journal of Vascular Nursing*, *36*(2), 64-70.
